# Base editing effectively prevents early-onset severe cardiomyopathy in *Mybpc3* mutant mice

**DOI:** 10.1038/s41422-024-00930-7

**Published:** 2024-02-09

**Authors:** Shuo Wu, Ping Yang, Zilong Geng, Yige Li, Zhizhao Guo, Yingmei Lou, Shasha Zhang, Junhao Xiong, Huan Hu, Xiaoling Guo, William T. Pu, Yan Zhang, Dan Zhu, Bing Zhang

**Affiliations:** 1 Key Laboratory of Systems Biomedicine, Shanghai Center for Systems Biomedicine, Department of Cardiovascular Surgery, Shanghai Chest Hospital, Engineering Research Center of Techniques and Instruments for Diagnosis and Treatment of Congenital Heart Disease, Institute of Developmental and Regenerative Medicine, Xin Hua Hospital, School of Medicine, Shanghai Jiao Tong University, Shanghai, China; 2 Basic Medical Research Center, the Second Affiliated Hospital and Yuying Children’s Hospital of Wenzhou Medical University, Wenzhou, Zhejiang China; 3 Department of Cardiology, Boston Children’s Hospital, Harvard Medical School, Boston, MA USA; 4 Harvard Stem Cell Institute, Harvard University, Boston, MA USA; 5 School of Biomedical Engineering, Shanghai Jiao Tong University, Shanghai, China

**Keywords:** Genomic analysis, Single-strand DNA breaks

Dear Editor,

Hypertrophic cardiomyopathy (HCM) is a primary myocardial disorder featured by left ventricular (LV) hypertrophy and cardiac dysfunction with an estimated global morbidity of 1:200–1:500.^1^ HCM has severe clinical manifestations including heart failure, arrhythmia, sudden cardiac death and stroke. Myosin-binding protein C3 (*MYBPC3*) mutations account for more than 40% pathogenic variants causing HCM.^2^ Homozygous or compound heterozygous truncating variants of *MYBPC3* are leading genetic causes of fetal and childhood-onset HCM characterized by heart failure and life-threatening ventricular arrhythmias.^3^ Currently available medications fail to rescue these infants. Base editing is a form of genome editing that enables direct conversion of individual nucleotides at specific genomic sites, without introducing a double-strand DNA break.^4,5^ Over the last few years, base editing has emerged as a powerful technology to change or generate genetic variants in a wide range of mitotic and postmitotic cells.^6–8^

To evaluate the potential of base editing to cure cardiomyopathy induced by homozygous *MYBPC3* premature termination codon mutation, using CRISPR-Cas9 technique, we generated a mouse model bearing *Mybpc3* c.2836 C > T (p.R946X) mutation (*Mybpc3*^R946X/R946X^) analogous to human *MYBPC3* p.R943X mutation that resulted in an early termination at amino acid 946 (Supplementary information, Fig. S1a, b).^9,10^ Sanger sequencing validated the correct installation of c.2836 C > T to exon 27 in the mouse genome (Supplementary information, Fig. S1c). Mybpc3 protein was undetectable in A-bands of *Mybpc3*^R946X/R946X^ cardiomyocytes (Supplementary information, Fig. S1d, e). We further performed serial echocardiography and observed LV hypertrophy with increased thickness of ventricular free wall in *Mybpc3*^R946X/R946X^ mice (Supplementary information, Fig. S2a). The LV internal diameter at diastole and systole increased significantly from the age of 2 months. In accordance with the chamber dilation, ejection fraction (EF), a measure of systolic function, declined to ~1/3 of that of the wild-type (WT) mice at 2 months of age. Although heart function declined at an early stage, *Mybpc3*^R946X/R946X^ mice survived for over 12 months.

Furthermore, we performed a postmortem examination at the age of 6 months. Compared to *Mybpc3*^R946X/+^ and *Mybpc3*^WT^, *Mybpc3*^R946X/R946X^ hearts were significantly larger with heart weight-to-tibia length (HW/TL) ratio being ~2-fold over that of the WT hearts (Supplementary information, Fig. S2b, c). Coronal sections illustrated the enlarged LV chamber, thickened myocardial walls and myofiber disarray of *Mybpc3*^R946X/R946X^ hearts (Supplementary information, Fig. S2d, e). Cardiomyocyte area revealed by wheat germ agglutinin (WGA) staining was significantly greater in *Mybpc3*^R946X/R946X^ mice, indicating the occurrence of cardiomyocyte hypertrophy (Supplementary information, Fig. S2f, g). Excessive interstitial and perivascular collagen deposition was also observed in 12-week-old *Mybpc3*^R946X/R946X^ mouse hearts, along with elevated expression of hypertrophic markers *Acta1* and *Nppa* (Supplementary information, Fig. S2h–j). We further measured the cardiac surface electrocardiogram (ECG) and revealed consistently prolonged QRS interval in the *Mybpc3*^R946X/R946X^ hearts indicating LV hypertrophy (Supplementary information, Fig. S2k, l). Overall, these results demonstrated that introducing the homozygous *Mybpc3*^R946X/R946X^ mutation leads to cardiomyopathy in neonatal mice that recapitulates key features of patients with biallelic *MYBPC3*-truncating variants.^9^

To correct *Mybpc3* c.2836 C > T mutation, we employed an adenine base editor (ABE) that catalyzes A·T to G·C conversion. Among the reported ABEs, ABEmax with its TadA-7.10 adenine deaminase has a narrow editing window, relatively high on-target activity and low bystander activity (Supplementary information, Fig. S3a).^5^ SpCas9 nickase used in ABEmax has an NGG protospacer adjacent motif (PAM), which precludes the c.2836 C > T mutation from being positioned within the editing window. To circumvent this restriction, we used SpRY-ABEmax which can target nearly all PAMs by replacing SpCas9 nickase with its PAMless variant SpRYCas9 nickase (Supplementary information, Fig. S3b).^11^ To edit mutant A on the bottom target DNA strand (A6 in sgRNA1) specifically and avoid including other adenines (A2 and A8 in sgRNA1), we designed two sgRNAs (sgRNA1&2) with CCC and GCC PAMs respectively (Supplementary information, Fig. S3a). Since SpRYCas9 is predicted to favor TGC PAM over NCN PAMs, we added another sgRNA (sgRNA3) with TGC PAM, although this moved the mutant A (A8 in sgRNA3) one position out of the high-activity window.

To evaluate editing efficiency of SpRY-ABEmax in vitro, we isolated mouse embryonic fibroblasts (MEFs) from *Mybpc3*^R946X/R946X^ embryos and used lentivirus to efficiently introduce SpRY-ABEmax (Supplementary information, Fig. S3d). Since in vivo delivery would utilize adeno-associated virus (AAV), and SpRY-ABEmax (6.5 kb) exceeds the capacity of a single AAV, we split SpRY-ABEmax into two parts using trans-splicing intein (Supplementary information, Fig. S3c).^6^ The N- and C-terminal parts of SpRY-ABEmax were expressed in MEF cells, and 35% of them spontaneously assembled into full-length SpRY-ABEmax (Supplementary information, Fig. S3e). High-throughput sequencing (HT-seq) showed that the editing efficiencies of three sgRNAs were 0.21% ± 0.02%, 4.74% ± 1.18% and 4.53% ± 1.77% respectively (Supplementary information, Fig. S3f). All three guide RNAs displayed low bystander editing efficiencies at other adenines in the protospacer except A4 in the sgRNA3 (Supplementary information, Fig. S3f).

The insufficient *Mybpc3* mutation correction of SpRY-ABEmax compelled us to develop an editor with superior editing activity. TadA-8e-V106W is an artificially evolved deaminase with higher editing activity than TadA-7.10 along with low RNA editing activty.^12^ We fused TadA-8e-V106W with SpRYCas9 and named it SpRY-ABE8e (Supplementary information, Fig. S4a, b). We used the same sgRNAs (sgRNA1–3) and trans-splicing intein strategy to express SpRY-ABE8e in *Mybpc3*^R946X/R946X^ MEFs, and 32% of them assembled into full-length SpRY-ABE8e (Supplementary information, Fig. S4c). Compared to SpRY-ABEmax, SpRY-ABE8e with sgRNA2 and sgRNA3 demonstrated significantly higher on-target editing efficiency (Supplementary information, Fig. S4d).

The bystander editing (A2 in sgRNA1) of SpRY-ABE8e also increased and altered the encoded amino acid from valine (V) to alanine (A), which share nonpolar and aliphatic traits. Structure prediction using AlphaFold2 indicated that the V to A substitution did not change MYBPC3 3D structure (Supplementary information, Fig. S5a). Off-target editing is another potential complication of base editing. To examine the off-target editing of SpRY-ABE8e, we selected 17 top-ranked off-target sites containing 81 to 103 editable adenines as predicted by Cas-OFFinder for each sgRNA and performed HT-seq (Supplementary information, Fig. S6a). SgRNA2 and sgRNA3 demonstrated low off-target editing activities at all the tested adenine sites except one, while sgRNA1 had high off-target editing activity at 4 adenine sites (Supplementary information, Fig. S5b). Based on these results, sgRNA2 was chosen for the following in vivo experiments.

For efficient delivery of SpRY-ABE8e to the cardiomyocytes in vivo, we packaged it as split inteins into AAV9 (Fig. 1a). Cardiomyocyte-specific expression was driven by the chicken cardiac troponin T (cTnT) promoter. We injected two doses of AAV-SpRY-ABE8e (low: 0.5 × 10^14^ vg/kg per AAV; high: 1 × 10^14^ vg/kg per AAV) to evaluate efficacy and dose-response relationship. AAV9 was administered by subcutaneous injection into *Mybpc3*^R946X/R946X^ mice at postnatal (P) days 0 to 3. Saline was served as the vehicle control (Fig. 1b).

**Fig. 1:**
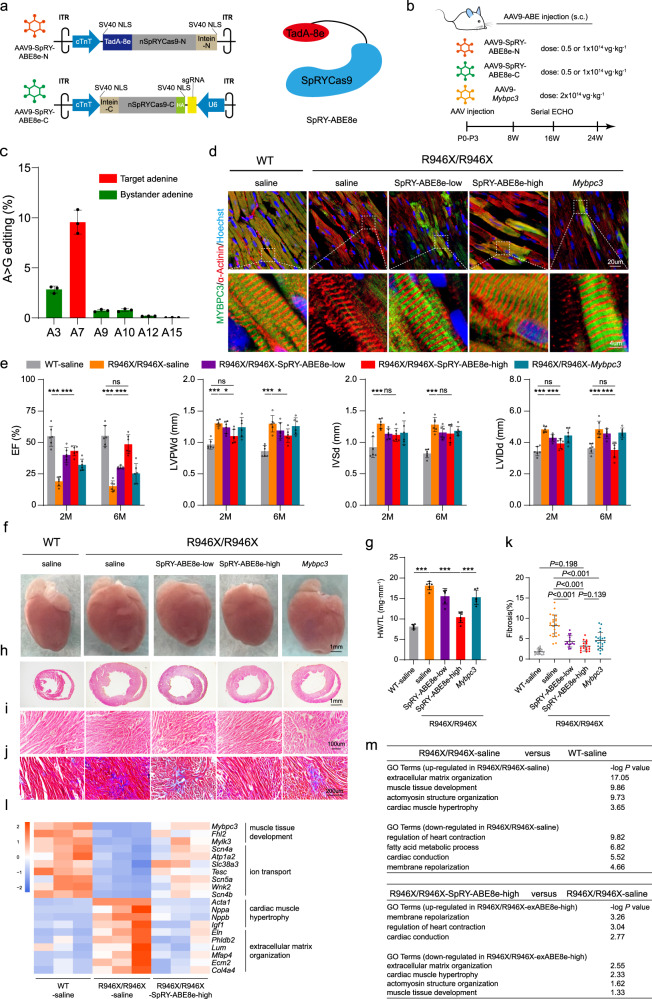
SpRY-ABE8e corrected *Mybpc3* mutation and prevented cardiomyopathy in a dose-dependent manner in vivo. **a** Schematic diagram of the dual AAV-SpRY-ABE8e system. **b** Experimental design for in vivo therapy using AAV-SpRY-ABE8e or *Mybpc3* gene replacement. **c** Editing efficiency in the hearts 6 months after high-dose SpRY-ABE8e treatment. *n* = 3. Data represent means ± SD. **d** IF staining showing the proper expression and assembly of MYBPC3 in the sarcomere of hearts of mice treated with AAV-SpRY-ABE8e or AAV-*Mybpc3*. **e** Serial echocardiography analysis of *Mybpc3*^R946X/R946X^ mice treated with low-dose or high-dose of AAV-SpRY-ABE8e or AAV-*Mybpc3*. *n* = 6 for each group. Data represent means ± SD and tested with two-way ANOVA followed by Holm-Sidak’s post hoc test. *P < 0.05; **P < 0.01; ***P < 0.001; ns, not significant. **f** Representative anatomic images of AAV-SpRY-ABE8e- and AAV-*Mybpc3*-treated hearts 6 months post injection. **g** HW/TL ratio 6 months post injection. *n* = 6 for each group. Data represent means ± SD and tested with one-way ANOVA followed by Tukey post hoc test. ***P < 0.001. **h** H&E staining of the heart sections from saline-, AAV-SpRY-ABE8e- or AAV-*Mybpc3*-treated mice 6 months post injection. **i** High-dose SpRY-ABE8e, but not low dose SpRY-ABE8e or AAV-*Mybpc3*, alleviated myofiber disarray. **j**, **k** Masson trichrome staining revealed reduced fibrosis in all AAV-treated groups (**j**). The fibrotic region in blue was quantified with Image J (**k**). 3 slices per heart from 5 hearts were calculated for each group. Data represent means ± SD and tested with one-way ANOVA followed by Tukey post hoc test. **l** Heat map visualization of RNA-seq data showing that heart failure- and collagen-related genes were downregulated in high-dose SpRY-ABE8e-treated hearts compared with saline-treated hearts in *Mybpc3*^R946X/R946X^ mice. **m** Gene ontology (GO) analyses of differentially expressed genes (DEGs) between saline-treated *Mybpc3*^R946X/R946X^ hearts and saline-treated WT hearts (top), and DEGs between high-dose SpRY-ABE8e-treated and saline-treated *Mybpc3*^R946X/R946X^ hearts (bottom).

Six months after the administration, high-dose SpRY-ABE8e introduced an average of 9.56% A > G transition at the target adenine (Fig. 1c), while 4.64% by low-dose injection (Supplementary information, Fig. S7c). Since cardiomyocytes only account for ~25%–35% cells in the heart,^13^ the editing efficiency of the high-dose SpRY-ABE8e was estimated to be ~30% in cardiomyocytes. Consistent with this estimation, immunofluorescence (IF) staining demonstrated that high-dose SpRY-ABE8e recovered MYBPC3 protein in 32.2% ± 8.5% cardiomyocytes (Supplementary information, Fig. S7d). The majority of the corrected cardiomyocytes were clustered rather in a single form, indicating that they bear the proliferative potential (Supplementary information, Fig. S7e). In corrected cells, MYBPC3 localized appropriately to sarcomere A-bands (Fig. 1d). α-Actinin expression was higher and more aligned in the corrected cardiomyocytes than in the uncorrected ones (Supplementary information, Fig. S7f). We observed low bystander editing activity compared to on-target editing (Fig. 1c). Moreover, over 99% of the bystander editing happened together with the on-target editing (Supplementary information, Fig. S7b). We also measured genome editing induced by high-dose SpRY-ABE8e in liver, lung, spleen and quadriceps muscle and found very low editing frequencies, indicating that the editing is cardiomyocyte specific (Supplementary information, Fig. S7g).

Unexpectedly, high-dose SpRY-ABE8e treatment restored 78%–110% of MYBPC3 protein expression, and low-dose SpRY-ABE8e treatment restored 38%–70% of MYBPC3 protein, which were higher than the ratio of gene correction (Supplementary information, Fig. S7h, i). We reasoned that this discrepancy was possibly due to transcriptional activation or enhanced protein stability in the gene-corrected cells, which had been observed in several in vivo base editing settings.^6,14,15^ To compare with AAV9-mediated gene replacement, we also treated *Mybpc3*^R946X/R946X^ mice with AAV-*Mybpc3* at the same dose to high-dose SpRY-ABE8e (Fig. 1b) and the AAV-*Mybpc3* treatment only recovered 41.4% ± 4.6% of MYBPC3 protein (Supplementary information, Fig. S7h, i). Episomal expression of MYBPC3 may be lost or diluted during the neonatal cardiomyocyte growth, but which was refrained from the SpRY-ABE8e-mediated genomic correction. Although the mechanism remains unsolved, our results indicate that base editing possesses a greater potential over gene replacement to restore MYBPC3 expression in the *Mybpc3*^R946X/R946X^ hearts at neonatal stage.

In agreement with high MYBPC3 recovery, serial echocardiography illuminated that high-dose SpRY-ABE8e significantly improved systolic heart function, chamber dilation and wall thickening from the age of 2 months to 6 months in *Mybpc3*^R946X/R946X^ mice (Fig. 1e; Supplementary information, Fig. S8a). Low-dose SpRY-ABE8e also significantly improved systolic function but not the chamber diameter and wall thickness, and similar results were found with AAV-*Mybpc3*. Of note, the beneficial effects of low-dose SpRY-ABE8e and AAV-*Mybpc3* on EF declined at 6 months compared to 2 months, whereas high-dose SpRY-ABE8e’s efficacy was retained if not improved at 6 months. In consistent with functional improvement, high-dose SpRY-ABE8e significantly attenuated QRS interval prolongation (Supplementary information, Fig. S8b, c). Together, these results underscore the importance of MYBPC3 protein recovery that accounts for preventing pathological remodeling in *Mybpc3* mutant mice.

Furthermore, postmortem analysis showed that high-dose but not low-dose SpRY-ABE8e or AAV-*Mybpc3* prevented cardiac enlargement, which was further confirmed by HW/TL ratio and short axis of histological sections (Fig. 1f–h). The disarray of muscle bundles was improved as well (Fig. 1i). In comparison, low-dose SpRY-ABE8e and AAV-*Mybpc3* improved the bundle disarray, ventricular dilation, and wall thickness to a limited extent. WGA staining revealed that low-dose SpRY-ABE8e, high-dose SpRY-ABE8e and AAV-*Mybpc3* all reduced cardiomyocyte cross-sectional area (Supplementary information, Fig. S8d, e). All three treatments reduced fibrosis, especially high-dose SpRY-ABE8e, which reduced fibrosis by 61.5% compared to saline treatment (Fig. 1j, k; Supplementary information, Fig. S8f). Together, these assays demonstrated that high-dose base editor offered significantly better therapeutic effects than AAV-*Mybpc3*, suggesting a greater potential of base editing as a strategy to prevent cardiomyopathy.

To further examine transcriptomic changes of *Mybpc3*^R946X/R946X^ hearts after base editing, we performed bulk RNA-seq and identified 727 genes differentially expressed between *Mybpc3*^R946X/R946X^ and WT hearts (Supplementary information, Fig. S9a, b). The transcriptomic profile of SpRY-ABE8e*-*treated *Mybpc3*^R946X/R946X^ hearts became more like WT hearts compared with saline-treated *Mybpc3*^R946X/R946X^ hearts. GO analysis showed that functions of these DEGs were associated with extracellular matrix deposition, muscle development and hypertrophy, and cardiac conduction (Fig. 1m). Comparing high-dose SpRY-ABE8e to saline treatment identified 137 DEGs. These genes had overlapping functions to genes differentially expressed between the mutant and WT mice, indicating that the same class of genes remained affected. Further exploration of RNA-seq data revealed a decrease in heart failure genes including *Acta1*, *Nppa*/*Nppb*, and collagen-related genes including *Col4a4* and *Ecm2*; as well as a recovery of cardiac development- and conduction-related genes after SpRY-ABE8e treatment (Fig. 1l). The expression of *Acta1* and *Nppa* was also reduced after low-dose SpRY-ABE8e and *Mybpc3* treatments while the changes were more significant in high-dose SpRY-ABE8e-treated mice (Supplementary information, Fig. S9c). These results show that SpRY-ABE8e base editing ameliorates the dysregulated gene expression in *Mybpc3*^R946X/R946X^ hearts.

To assess off-target editing by SpRY-ABE8e, we isolated genomic DNA from the hearts and measured off-target editing at 17 sites analyzed in MEFs. In agreement with in vitro results, dual AAV-SpRY-ABE8e introduced nearly background off-target editing in hearts (Supplementary information, Fig. S9d). To assess potential RNA deaminase activity of SpRY-ABE8e, we analyzed the RNA-seq data and detected minimal and comparable adenine-to-inosine editing of mRNA in high-dose SpRY-ABE8e-treated hearts compared to the saline-treated ones (Supplementary information, Fig. S9e). These results demonstrate that our dual AAV9-SpRY-ABE8e system has negligible DNA and RNA off-target activities in the hearts.

In this study, we generated a transgenic murine model bearing a human *MYBPC3*-truncating variant that developed early-onset, severe cardiomyopathy with rapid evolution to cardiac dysfunction, recapitulating key features of patients with biallelic *MYBPC3* pathogenic variants. We developed a potent, PAM-extended dual base editor that efficiently and precisely corrected the *Mybpc3* nonsense mutation, thereby preventing cardiac hypertrophy and dysfunction in the mutant mouse model. In sum, our study, in line with others demonstrates the great potential of base editing as a new therapeutic modality to treat genetic cardiomyopathy, which lays out a foundation for future applications in clinics.^7,8^

### Supplementary information

Supplementary Table S1

Supplementary Table S2

Supplementary Table S3

Supplementary information

Supplementary Figure S1

Supplementary Figure S2

Supplementary Figure S3

Supplementary Figure S4

Supplementary Figure S5

Supplementary Figure S6

Supplementary Figure S7

Supplementary Figure S8

Supplementary Figure S9

## Data Availability

DNA sequencing files can be accessed at the National Center for Biotechnology Information Sequence Read Archive (NCBI SRA) with the accession code PRJNA1000763. Raw and processed RNA-seq data are deposited to the Gene Expression Omnibus (GEO) database and available with the accession number GSE239872.
