# Supplementary material for: Base editing effectively prevents early-onset severe cardiomyopathy in Mybpc3 mutant mice
Source: Cell Res. 2024 Feb 9;34(4):327–30. doi: 10.1038/s41422-024-00930-7 (PMC10978934; doi:10.1038/s41422-024-00930-7)
Supplement: Supplementary file 4 — Supplementary information [file 41422_2024_930_MOESM4_ESM.docx]

**Supplementary information**

**Base editing effectively prevents the early-onset severe cardiomyopathy in Mybpc3 mutant mice**

**Running Title: Genome correction prevents the hereditary cardiomyopathy**

Shuo Wu^1†^, Ping Yang^1†^, Zilong Geng^1†^, Yige Li^1†^, Zhizhao Guo^1^, Yingmei Lou^1^, Shasha Zhang^1^, Junhao Xiong^1^, Huan Hu^1^, William T. Pu^2,3^, Yan Zhang^4*^, Dan Zhu^1*^, Bing Zhang^1*^

^1^Key Laboratory of Systems Biomedicine, Shanghai Center for Systems Biomedicine, Department of Cardiovascular Surgery, Shanghai Chest Hospital, Department of Pediatric Cardiology, Institute of Developmental and Regenerative Medicine, Xin Hua Hospital, School of Medicine, Shanghai Jiao Tong University, Shanghai, China

^2^Department of Cardiology, Boston Children’s Hospital, Harvard Medical School, MA, USA

^3^Harvard Stem Cell Institute, Harvard University, MA, USA

^4^School of Biomedical Engineering, Shanghai Jiao Tong University, Shanghai,200127, China

^*^Corresponding authors:

E-mail addresses: [bingzhang@sjtu.edu.cn](mailto:bingzhang@sjtu.edu.cn) (Bing Zhang), [yanzhang@sjtu.edu.cn](mailto:yanzhang@sjtu.edu.cn) (Yan Zhang), [zhudanmd@163.com](mailto:zhudanmd@163.com) (Dan Zhu),

^†^These authors contributed equally: Shuo Wu, Ping Yang, Zilong Geng, Yige Li.

**Materials and Methods**

**Experimental Animals**

*Mybpc3*^R946X/+^ knock-in mouse was generated in GemPharmatech China using CRSIPR-Cas9 technology (Fig. S1B). In brief, Cas9 mRNA, *Mybpc3* targeting sgRNA and a donor template containing *Mybpc3* c.2836C>T substitution were injected into mouse zygotes. Positive mutation insertion of F_0_ pups were validated by Sanger sequencing and further bred to wild type C57BL/6J mice for stable transmission. Genotyping was performed by amplification-refractory mutation system (ARMS-PCR) using two pairs of primers: for WT allele (forward: ACGGCTGCTGTTCCGAGTAC, reverse: GAGAGCCCAAGGCATAGATG AAG) and for mutant allele (forward: CACGGCTGCTGTTCTGAGTAA, reverse: GAGA GCCCAAGGCATAGATGAAG). All animal procedures were in accordance with animal protocols approved by the Institutional Animal Care and Use Committee of Shanghai Jiao Tong University.

**Cloning**

For lentiviral vector cloning, the SpRY-ABEmax coding sequence was cloned from pCMV-T7-ABEmax (7.10)-SpRY-P2A-EGFP (Addgene #140003),^1^ divided into two parts at Cys 574 of SpRYCas9 as shown in Fig. S3c and then inserted into lentiCRISPRv2 backbone (Addgene #52961) after fused with split intein from *Nostoc* *punctiforme* (Npu).^2^ The sgRNAs were inserted into the C-terminal part under the control of U6 promoter. For the construction of ­SpRY-ABE8e, TadA-7.10 was replaced with TadA-8e-V106W synthesized in GENEWIZ.^3^

For AAV vector cloning, the SpRY-ABEmax and ­SpRY-ABE8e were amplified by PCR from the lentivirus vectors aforementioned and cloned into pX601-AAV-CMV::NLS-SaCas9-NLS-3xHA-bGHpA;U6::BsaI-sgRNA (addgene #61591) together with a chicken cTnT promoter for specific expression in cardiomyocyte.^4-6^ A truncated woodchuck hepatitis virus post transcriptional regulatory element (WPRE)-W3, were inserted into the downstream of SpRY-ABEmax and ­SpRY-ABE8e to enhance the transgene expression.^2^

**Cell culture**

MEF cells were harvested from E13.5 *Mybpc3*^R946X/R946X^ embryos as described previously.^7^ In brief, the embryos were dissected out of the uterus and immediately transferred to a 10 cm cell culture filled with ice-cold PBS. After removing the head, liver and limbs, the body was minced with a razor blade and then digested with 1ml 2.5% trypsin for 30-45 minutes at 37°C. 4ml of Dulbecco’s Modified Eagle Medium (DMEM) containing 10% fetal bovine serum (FBS) was added to the reaction to deactivate the trypsin. The tissues were pipetted vigorously and centrifuged at 1000 RPM for 5 minutes. The cell pellet was resuspended in DMEM with 10% FBS for further cell expansion.

HEK293 and HEK293T cells were regularly cultured in DMEM supplemented with 10% FBS at 37°C with 5% CO_2_.

**Lentivirus generation and transduction**

The lentivirus production and transduction were carried out as previously described.^8^ Lenti-ABE vectors, along with VSV-G expressing envelop vector pMD2.G and packaging vectors psPAX2, were transfected into HEK293T cells using polyethylenimine (PEI, Polysciences) to generate virus particles. Lentiviral particles were purified from the cell culture medium using 9% PEG6000 in 0.3M NaCl, resuspended in PBS and stocked in -80˚C until use.

MEF cells cultured in 6-well plates were pretreated with 8 μg/ml hexadimethrine bromide (Sigma-Aldrich, H9268) and then transduced with lentivirus in two to three multiplicities of infection (MOI). The cells were maintained for 20 days before subjected to the genomic DNA and protein extraction.

**AAV generation and administration**

The procedure of AAV9 virus production was described previously.^4^ AAV-ABE vectors were transfected into HEK293T cells together with two helper plasmids, Rep/Cap and pHGTI/delta, using 2µg/ml PEI. 60 hours after transfection, cells were collected and resuspended in lysis buffer (20 mM Tris pH 8.0, 150 mM NaCl, 1 mM MgCl2) supplemented with 250 U/mL Benzonase (20157ES, YEASEN) at 37˚C for 15 min in order to remove the cellular DNA. AAV9 viruses were purified using iodixanol (D1556, Sigma-Aldrich)-gradient ultracentrifugation (48,000 RPM for 90 min), and then buffer-exchanged and concentrated with Amicon 100kD columns (Millipore, UFC9100). Purified AAV9 viruses were suspended in PBS, aliquoted and stored at -80°C until use. The viral titer was determined by quantifying the copies of viral genome using qPCR with primers specific for ITR region and promoter region (sequences in table 1).

5×10^13^ vg·kg^-1^ or 1×10^14^ vg·kg^-1^ virus was subcutaneously injected to P0-P3 *Mybpc3*^R946X/R946X^ mice for *in vivo* genomic editing.

**Echocardiography**

Transthoracic echocardiography was executed using a Visual Sonics 3100 system with an MS-550S (40 MHz) transducer. Mice were anesthetized with 2% isoflurane inhalation, and the heart rate was stabilized between 450-550 beats per minute before test. Two-dimensional B-Mode imaging was used to visualize the left ventricle and the aortic outflow tract. A number of parameters including fraction shortening (FS), ejection fraction (EF), left ventricular end-diastolic internal diameter (LVIDd), left ventricular end-systolic internal diameter (LVIDs), left ventricular end-diastolic posterior wall thickness (LVPWd), left ventricular end-systolic posterior wall thickness (LVPWs), end-diastolic interventricular septum (IVSd), and end-systolic interventricular septum (IVSs) were calculated from at least three distinct frames for each mouse.

**Histopathology**

**IHC staining**

The mouse heart was perfused with a 0.1% 2,3-Butanedione monoxime/PBS solution through the aorta and then fixed in a 4% paraformaldehyde/PBS solution for 24 hours at 4°C. For paraffin embedding, the fixed hearts were dehydrated using a gradient of ethanol, cleared with xylene twice, and embedded in paraffin for overnight. 10μm coronal or cross sections were stained with Hematoxylin & Eosin (YEASEN, 60524ES60) or Masson’s Trichrome (YEASEN, 60532ES) according to the manufacturer’s protocol. The images were captured with 200X microscope and measured with ImageJ software.

**Immunofluorescences**

Cryosections in 6 µm were air-dried, washed with PBS, and subjected to antigen retrieval by heating in citrate buffer (0.01M Citric Acid anhydrous, 0.05% Tween-20) for 30 minutes. After cooling down to room temperature, sections were blocked with blocking buffer (5% normal donkey serum, 0.1% TritonX-100 in PBS) for 30 min. Primary antibodies, including anti-MYBPC3 (Sigma-Aldrich, HPA043898, 1:500) and anti-α-Actinin (R&D system, AF8279, 1:200), diluted in dilution buffer (5% normal donkey serum and 0.3% TritonX-100 in PBS), were added to sections and incubated overnight at 4°C. After washing with PBST (0.1% Tween20/PBS) for three times, sections were then incubated with Alexa Fluor-conjugated donkey-derived secondary antibodies (1: 200, Thermo Fisher Scientific) for 1 hour at room temperature. Hoechst 33342 (Thermo Fisher Scientific, 62249) was used to label the cellular nuclei. Fluorescent images were captured with Nikon A1Si confocal microscope. For the calculation of correct cardiomyocytes ratio, we counted the number of cells co-staining with α-Actinin and MYBPC3 and cells staining with α-Actinin from total 18 slides of 6 treated mice for each group. We further counted the number of correct cells in a single form and correct cells next to other correct cells from total 18 slides of 6 high-dose SpRY-ABE8e treated mice. For the evaluation of the expression of α-Actinin, we calculated the fluorescence intensity of α-Actinin from correct cells or incorrect cells by ImageJ software from total 18 slides of 6 high-dose SpRY-ABE8e treated mice. The intensity was normalized by average intensity of incorrect cells for each slide.

WGA staining was also performed with paraffin sections. Briefly, the sections were dewaxed with xylene and then rehydrated with a gradient of ethanol. WGA conjugated with Alexa Fluor 647 (Thermo Fisher Scientific, W32466) was resolved with a dilution buffer (5% normal donkey serum and 0.3% TritonX-100 in PBS) for a final concentration of 2 µg/ml and incubated with the sections for 30 minutes at room temperature. The cellular nuclei were stained with Hoechst 33342.

**Quantitative real-time PCR**

Total RNA was extracted from mouse hearts with RNAsimple Total RNA Kit (TIANGEN, DP419). 500 ng of RNA were reversed transcribed with HiScript II 1^st^ Strand cDNA Synthesis Kit (+gDNA wiper) (Vazyme, R212), and quantified in Roche LightCycler 96 instruments using ChamQ Universal SYBR qPCR Master Mix (Vazyme, Q711). GAPDH was deployed for normalization. All primers used here were documented in Table 1.

**Western blot**

Tissue samples were firstly pulverized in Tissue Lyser while cell samples not. Proteins were extracted with high-salt buffer (20 mM HEPES pH 7.9, 420 mM NaCl, 25% Glycerol, 0.2 mM EDTA, 0.5 mM DTT, 1% NP40, and 0.5% SDS) by incubating on ice for 30 minutes. The protein samples were quantified with BCA assay (Beyotime, P0010), separated with SDS-PAGE, and probed with primary antibodies (anti-MYBPC3, Santa Cruz Biotechnology, sc-137180; anti-GAPDH, ABclonal, A19056; anti-HA, CST, 2367S; anti-CRISPR/Cas, Diagenode, C15310258) and further with HRP-conjugated secondary antibodies (1:200, Invitrogen) after wet transfer. The aimed proteins were revealed with LumiQ HRP Substrate solution kit (Share-Bio, SB-WB012) and detected in an Amersham Imager 800 and quantified with ImageJ.

**On target analysis**

Genomic DNA was extracted from collected cells or mice hearts with Lysis Buffer (50 mM Tris-HCl pH 8.0, 100 mM EDTA, 100 mM NaCl, 1% SDS, 0.5 mg·ml^-1^ proteinase K), followed by precipitation with isopropyl-ketone. The isolated DNA was further washed with 70% ethanol and diluted with TE buffer.

For high throughput sequencing of edited genomic DNA, the on-target and off-target sites were amplified using PrimeSTAR MAX Polymerase (Takara, R045) with primers including barcode sequences. The amplicons were constructed to libraries and sequenced on an Illumina NovaSeq 6000 platform in Novogene Corporation. The sequencing data was sorted into different groups using fastq-multx (https://github.com/brwnj/fastq-multx) based on the barcodes embedded in the PCR primers, and editing efficiency of each site was analyzed using CRISPResso2 (https://github.com/pinellolab/CRISPResso2) following the standard protocol.^9^

**Off-target and bystander analysis**

Cas-OFFinder 2.4 (http://www.rgenome.net/cas-offinder/) was deployed to predict off-target sites with different PAM sequences and sgRNAs.^10^ The off-target candidates were ranked based on the number of mismatches and bulge sizes. For each sgRNA, 8 sites with NRN PAM and 4 sites with NYN PAM were selected for target sequencing. Because PAM-less SpRYCas9 is vulnerable for vector self-editing, we also tested another 5 potential off-target sites result from de novo sgRNAs created by vector self-editing for both NRN and NYN PAM.^11^ All the testing primers were documented in Table1. The purified DNA was subjected to high throughput sequencing and analyzed with CRISPResso2 as described above.

**RNA-seq and analysis**

Total RNA was extracted with RNAsimple Total RNA Kit (TIANGEN, DP419). mRNA was enriched using oligo(dT)-attached magnetic beads. The complementary DNA (cDNA) was synthesized after mRNA fragmentation, constructed into barcoded library and sequenced in an MGISEQ2000 platform at BGI Genomics China.

The FASTQ files were aligned to mm10 genome by hisat2 (http://daehwankimlab.github.io/hisat2/) after examined by FastQC.^12^ We utilized HTSeq (https://htseq.readthedocs.io/en/master/) for the quantification of mRNA abundance, and normalized the data using the Counts Per Million (CPM) method.^13^ DEseq2 (https://bioconductor.org/packages/release/bioc/html/DESeq2.html) was employed to identify differentially expressed genes (DEG) with adjusted *P* value <0.05. DAVID functional annotation tools (https://david.ncifcrf.gov/) were used to analyze gene ontology (GO).

A-to-I editing analysis on adenosines in the RNA-seq was calculated as a previously reported strategy.^14^ REDItools2 (https://github.com/BioinfoUNIBA/REDItools2) was employed to quantify the A-to-I editing percentage.^15^ Only adenosines with read coverage more than 10 and mapping or read quality score above 25, were considered and subjected to the calculation of A-to-I editing percentage in each sample.

**Statistical analysis**

Statistical analysis was performed by GraphPad Prism 9. All data are presented as mean ± standard deviation (SD). Shapiro-Wilk test was used to assess data normality, and *P* ≥ 0.05 was considered as a normal distribution. One-way ANOVA with the Tukey test was used to compare data containing more than two groups with normally distributed, while nonparametric Kruskal-Wallis test with Dunn multiple-comparisons test for non-normal distributed data. Two-way ANOVA with post hoc Holm-Sidak’s multiple comparison tests was performed for data with two independent variables. *P* < 0.05 was considered statistically significant.

**Supplementary discussion**

In this study, we generated a transgenic murine model bearing a human MYBPC3 truncating variant that developed early-onset, severe cardiomyopathy with rapid evolution to cardiac dysfunction, recapitulating key features of patients with biallelic *MYBPC3* pathogenic variants. We developed a potent, PAM-extended dual-AAV base editor systems that efficiently and precisely corrected the *Mybpc3* nonsense mutation, thereby preventing cardiac hypertrophy and dysfunction in the mutant mouse model. Our study demonstrates the immense potential of base editing to treat cardiac diseases including inherited cardiomyopathies.

*MYBPC3* c.2827C>T (p. R943X) is one of the most frequent HCM mutations, with an estimated 136,000 affected individuals globally. The pathogenicity of this truncating variant was not previously modeled in animals. This variant results in nonsense mediated decay and complete loss of cardiomyocyte *Mybpc3* RNA and protein, confirming a loss-of-function first proposed from iPSC-derived cardiomyocytes.^16^ *Mybpc3*^R946X/R946X^ mice developed rapid onset cardiomyopathy that quickly progressed to systolic dysfunction. The time course, also observed in *Mybpc3* knockout^17^ and *Mybpc3^t/t^* mice^18^, is consistent with patients with biallelic MYBPC3 truncating variants and distinct from heterozygous MYBPC3 truncating variants, which typically cause slowly progressive HCM with enhanced systolic function that progresses to systolic dysfunction late in the disease course.^19^ However, both homozygous and heterozygous R946X mutant mice survive over 12 months while human patients with biallelic truncating pathogenic mutations in MYBPC3 died within the first few months of life. The lack of infantile mortality was also observed by other Mybpc3 deficient mouse model and is worthy of further investigation.^17,18^ Although Mavacamten, a small molecule cardiac myosin inhibitor, was recently FDA-approved for treatment of HCM, it is restricted to patients with increased or preserved systolic function and therefore not applicable to most patients with biallelic *MYBPC3* variants.^20^ Our study demonstrated that genomic correction of R946X mutation using base editing efficiently prevents hypertrophic remodeling and preserves systolic function, suggesting that it is a viable approach for these patients.

To increase on-target editing efficacy, we used a newly developed adenine deaminase TadA-8e to build SpRY-ABE8e.^3^ We verified that ­SpRY-ABE8e is superior to ­SpRY-ABEmax in its on-target editing frequency. However, ­SpRY-ABE8e also exhibits higher bystander activity which is now one of the big concerns for clinical application of base editor. In our case, SpRY-ABE8e introduced a bystander V947A substitution along with R946X correction. This V947A missense mutation doesn’t alter the structure of MYBPC-C7 domain (Supplementary information, Fig. S5a) and has not been reported in Clinvar database suggesting the harmless of this mutation. However, additional caveat and safety assessment still need to be casted, which is particularly important in the future translational studies. Compared to SpRY-ABE8e, SpRY-ABEmax had lower activity and a smaller window for bystander and off-target editing. Although it did not fulfill the needs of our study, in other contexts ABEmax achieved satisfactory therapeutic efficacy^14,21,22^. Recently, a more accurate ABE variant ABE9 was invented with a mere 1-2 nucleotide-wide editing window which could further minimize the bystander effect.^23^ With these new base editors being rapidly developed, selecting the right editor will become a key issue requring careful consideration to design base editing therapy.

To overcome the PAM constraint, we used SpRYCas9, a nearly PAMless SpCas9 variant, that has seldom been used for base editing due concerns about reduced efficacy and higher off-target editing.^1^ However, in our study we found that SpRYCas9-mediated base editing achieved high on-target and low off-target efficiency with the appropriate sgRNA. Although SpRYCas9 in theory has no PAM limitation, we found the PAM sequence affected editing efficiency greatly. SgRNA3 exhibited similar editing activity to sgRNA2 even though the target adenine was out of optimized editing window, likely due to a more favorable PAM. These results demonstrate that PAM screening needs to be included in designing the base editing strategy. Guide sequence is another critical tier to achieve optimal editing. From our experience, sgRNA affects all types of editing events and needs to be empirically fine-tuned. Thus, a comprehensive optimization process is required to achieve safe and efficient genome editing. A cost-effective assay such as our MEF-based assay is invaluable for these optimization steps.

We found that normalization of MYBPC3 protein level is critical for effective treatment of biallelic *Mybpc3* cardiomyopathy in consistent with a previously reported gene replacement strategy.^24^ 78%-110% MYBPC3 recovery rate from high dose ­exABE8e almost completely restored heart function and remodeling, whereas 38%-70% from low-dose ­SpRY-ABE8e only achieved a partial and transient recovery. These results suggest that effective therapy requires restoration of MYBPC3 to at least 70% of normal levels, which is consistent with a previous study that illustrated 40% reduction of MYBPC3 content is sufficient to induce abnormal myofilament mechanics.^25^

Via head-to-head comparison, our study demonstrated better efficacy of base editing over gene replacement regarding MYBPC3 protein recovery and therapeutic outcome. At the same dosage as AAV-*Mybpc3*, ­SpRY-ABE8e recovered 2-3 folds more MYBPC3 protein than AAV-*Mybpc3* and demonstrated more sustained and significant phenotypic improvement.^24^ We anticipate that the ABE approach could be further improved by using a smaller Cas effector such as SaCas9 or Cas12, which would enable delivery of ABE with a single virus rather than the dual vector split intein strategy used in this study.^23,26,27^ On the other hand, employment of new AAV variants such as MYOAAV to elevate the infection rate of cardiomyocyte could also help to increase the ultimate edition efficiency.^28^

In sum, our study together with others demonstrates the great potential of base editing as a new therapeutic modality to treat genetic cardiomyopathy, which lays out a foundation for future application in clinics.^21,29^

**References**

1 Walton, R. T., Christie, K. A., Whittaker, M. N. & Kleinstiver, B. P. Unconstrained genome targeting with near-PAMless engineered CRISPR-Cas9 variants. *Science* **368**, 290-296 (2020). <https://doi.org:10.1126/science.aba8853>

2 Levy, J. M. *et al.* Cytosine and adenine base editing of the brain, liver, retina, heart and skeletal muscle of mice via adeno-associated viruses. *Nat Biomed Eng* **4**, 97-110 (2020). <https://doi.org:10.1038/s41551-019-0501-5>

3 Richter, M. F. *et al.* Phage-assisted evolution of an adenine base editor with improved Cas domain compatibility and activity. *Nature biotechnology* **38**, 883-U884 (2020). <https://doi.org:10.1038/s41587-020-0453-z>

4 Yu, H. *et al.* LARP7 Protects Against Heart Failure by Enhancing Mitochondrial Biogenesis. *Circulation* **143**, 2007-2022 (2021). <https://doi.org:10.1161/CIRCULATIONAHA.120.050812>

5 Ran, F. A. *et al.* In vivo genome editing using Staphylococcus aureus Cas9. *Nature* **520**, 186-191 (2015). <https://doi.org:10.1038/nature14299>

6 Ma, H., Sumbilla, C. M., Farrance, I. K., Klein, M. G. & Inesi, G. Cell-specific expression of SERCA, the exogenous Ca2+ transport ATPase, in cardiac myocytes. *Am J Physiol Cell Physiol* **286**, C556-564 (2004). <https://doi.org:10.1152/ajpcell.00328.2003>

7 Zhang, F. *et al.* LARP7 Is a BRCA1 Ubiquitinase Substrate and Regulates Genome Stability and Tumorigenesis (vol 32, pg 107974, 2020). *Cell Reports* **32** (2020).

8 Chen, J. H. *et al.* VEGF amplifies transcription through ETS1 acetylation to enable angiogenesis. *Nature Communications* **8** (2017). <https://doi.org:ARTN> 383

10.1038/s41467-017-00405-x

9 Clement, K. *et al.* CRISPResso2 provides accurate and rapid genome editing sequence analysis. *Nature biotechnology* **37**, 224-226 (2019). <https://doi.org:10.1038/s41587-019-0032-3>

10 Bae, S., Park, J. & Kim, J. S. Cas-OFFinder: a fast and versatile algorithm that searches for potential off-target sites of Cas9 RNA-guided endonucleases. *Bioinformatics* **30**, 1473-1475 (2014). <https://doi.org:10.1093/bioinformatics/btu048>

11 Ren, Q. *et al.* PAM-less plant genome editing using a CRISPR-SpRY toolbox. *Nat Plants* **7**, 25-33 (2021). <https://doi.org:10.1038/s41477-020-00827-4>

12 Kim, D., Paggi, J. M., Park, C., Bennett, C. & Salzberg, S. L. Graph-based genome alignment and genotyping with HISAT2 and HISAT-genotype. *Nature biotechnology* **37**, 907-915 (2019). <https://doi.org:10.1038/s41587-019-0201-4>

13 Putri, G. H., Anders, S., Pyl, P. T., Pimanda, J. E. & Zanini, F. Analysing high-throughput sequencing data in Python with HTSeq 2.0. *Bioinformatics* **38**, 2943-2945 (2022). <https://doi.org:10.1093/bioinformatics/btac166>

14 Koblan, L. W. *et al.* In vivo base editing rescues Hutchinson-Gilford progeria syndrome in mice. *Nature* **589**, 608-+ (2021). <https://doi.org:10.1038/s41586-020-03086-7>

15 Flati, T. *et al.* HPC-REDItools: a novel HPC-aware tool for improved large scale RNA-editing analysis. *BMC bioinformatics* **21**, 353 (2020). <https://doi.org:10.1186/s12859-020-03562-x>

16 Seeger, T. *et al.* A Premature Termination Codon Mutation in MYBPC3 Causes Hypertrophic Cardiomyopathy via Chronic Activation of Nonsense-Mediated Decay. *Circulation* **139**, 799-811 (2019). <https://doi.org:10.1161/Circulationaha.118.034624>

17 Harris, S. P. *et al.* Hypertrophic cardiomyopathy in cardiac myosin binding protein-C knockout mice. *Circ Res* **90**, 594-601 (2002). <https://doi.org:10.1161/01.res.0000012222.70819.64>

18 McConnell, B. K. *et al.* Dilated cardiomyopathy in homozygous myosin-binding protein-C mutant mice. *J Clin Invest* **104**, 1235-1244 (1999). <https://doi.org:10.1172/JCI7377>

19 Zen, K. *et al.* Analysis of circulating apoptosis mediators and proinflammatory cytokines in patients with idiopathic hypertrophic cardiomyopathy: comparison between nonobstructive and dilated-phase hypertrophic cardiomyopathy. *Int Heart J* **46**, 231-244 (2005). <https://doi.org:10.1536/ihj.46.231>

20 Green, E. M. *et al.* A small-molecule inhibitor of sarcomere contractility suppresses hypertrophic cardiomyopathy in mice. *Science* **351**, 617-621 (2016). <https://doi.org:10.1126/science.aad3456>

21 Chai, A. C. *et al.* Base editing correction of hypertrophic cardiomyopathy in human cardiomyocytes and humanized mice. *Nat Med* **29**, 401-411 (2023). <https://doi.org:10.1038/s41591-022-02176-5>

22 McAuley, G. E. *et al.* Human T cell generation is restored in CD3delta severe combined immunodeficiency through adenine base editing. *Cell* **186**, 1398-1416 e1323 (2023). <https://doi.org:10.1016/j.cell.2023.02.027>

23 Chen, L. *et al.* Engineering a precise adenine base editor with minimal bystander editing. *Nat Chem Biol* **19**, 101-110 (2023). <https://doi.org:10.1038/s41589-022-01163-8>

24 Mearini, G. *et al.* Mybpc3 gene therapy for neonatal cardiomyopathy enables long-term disease prevention in mice. *Nature Communications* **5** (2014). <https://doi.org:ARTN> 5515

10.1038/ncomms6515

25 O'Leary, T. S., Snyder, J., Sadayappan, S., Day, S. M. & Previs, M. J. MYBPC3 truncation mutations enhance actomyosin contractile mechanics in human hypertrophic cardiomyopathy. *J Mol Cell Cardiol* **127**, 165-173 (2019). <https://doi.org:10.1016/j.yjmcc.2018.12.003>

26 Davis, J. R. *et al.* Efficient in vivo base editing via single adeno-associated viruses with size-optimized genomes encoding compact adenine base editors. *Nat Biomed Eng* **6**, 1272-1283 (2022). <https://doi.org:10.1038/s41551-022-00911-4>

27 Wu, T. *et al.* An engineered hypercompact CRISPR-Cas12f system with boosted gene-editing activity. *Nat Chem Biol* (2023). <https://doi.org:10.1038/s41589-023-01380-9>

28 Tabebordbar, M. *et al.* Directed evolution of a family of AAV capsid variants enabling potent muscle-directed gene delivery across species. *Cell* **184**, 4919-4938 e4922 (2021). <https://doi.org:10.1016/j.cell.2021.08.028>

29 Reichart, D. *et al.* Efficient in vivo genome editing prevents hypertrophic cardiomyopathy in mice. *Nat Med* **29**, 412-421 (2023). <https://doi.org:10.1038/s41591-022-02190-7>
