# Supplementary material for: Base editing effectively prevents early-onset severe cardiomyopathy in Mybpc3 mutant mice
Source: Cell Res. 2024 Feb 9;34(4):327–30. doi: 10.1038/s41422-024-00930-7 (PMC10978934; doi:10.1038/s41422-024-00930-7)
Supplement: Supplementary file 5 — Supplementary Figure S1 [file 41422_2024_930_MOESM5_ESM.pdf]

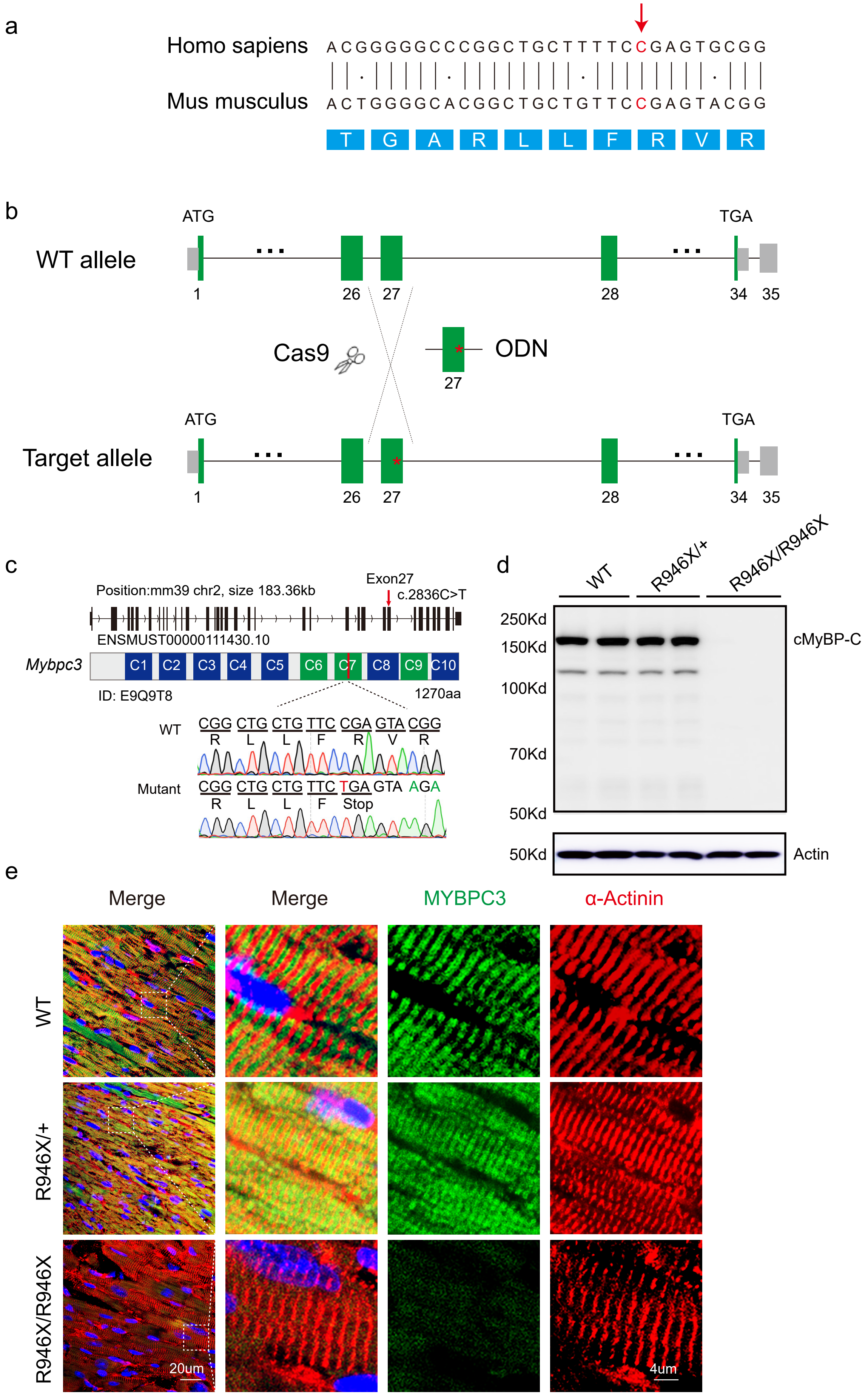

a
Homo sapiens
A C G G G G G C C C G G C T G C T T T T C C G A G T G C G G
Mus musculus
A C T G G G G C A C G G C T G C T G T T C C G A G T A C G G
T G A R L L F R V R
b
WT allele
ATG
TGA
1
26
27
28
34
35
Cas9
ODN
27
Target allele
ATG
TGA
1
26
27
28
34
35
c
Position:mm39 chr2, size 183.36kb
Exon27
c.2836C>T
ENSMUST00000111430.10
Mybpc3
C1 C2 C3 C4 C5 C6 C7 C8 C9 C10
ID: E9Q9T8
1270aa
WT
CGG CTG CTG TTC CGA GTA CGG
R L L F R V R
Mutant
CGG CTG CTG TTC TGA GTA AGA
R L L F Stop
d
WT
R946X/+
R946X/R946X
250Kd
150Kd
100Kd
70Kd
50Kd
50Kd
cMyBP-C
Actin
e
Merge
Merge
MYBPC3
α-Actinin
WT
R946X/+
R946X/R946X
20um
4um

Fig. S1. The construction of Mybpc3 p.R946X mouse model.

a Homology analysis of coding sequences around human *MYBPC3* c.2827C and mouse *Mybpc3* c.2836C.

b Generation diagram of *Mybpc3*^{R946X} transgenic mouse model using CRISPR-Cas9 technology.

c Validation of *Mybpc3*^{R946X} mutant mouse by Sanger sequencing.

d Western blot revealed MYBPC3 loss in *Mybpc3*$^{R946X/R946X}$ heart at the age of 3 months.

e Immunohistochemistry showed MYBPC3 loss in *Mybpc3*$^{R946X/R946X}$ heart sarcomeric A-bands at the age of 3 months. MYBPC3 (green), α-Actinin (red) and Hoechst (blue).
