# Supplementary material for: Base editing effectively prevents early-onset severe cardiomyopathy in Mybpc3 mutant mice
Source: Cell Res. 2024 Feb 9;34(4):327–30. doi: 10.1038/s41422-024-00930-7 (PMC10978934; doi:10.1038/s41422-024-00930-7)
Supplement: Supplementary file 6 — Supplementary Figure S2 [file 41422_2024_930_MOESM6_ESM.pdf]

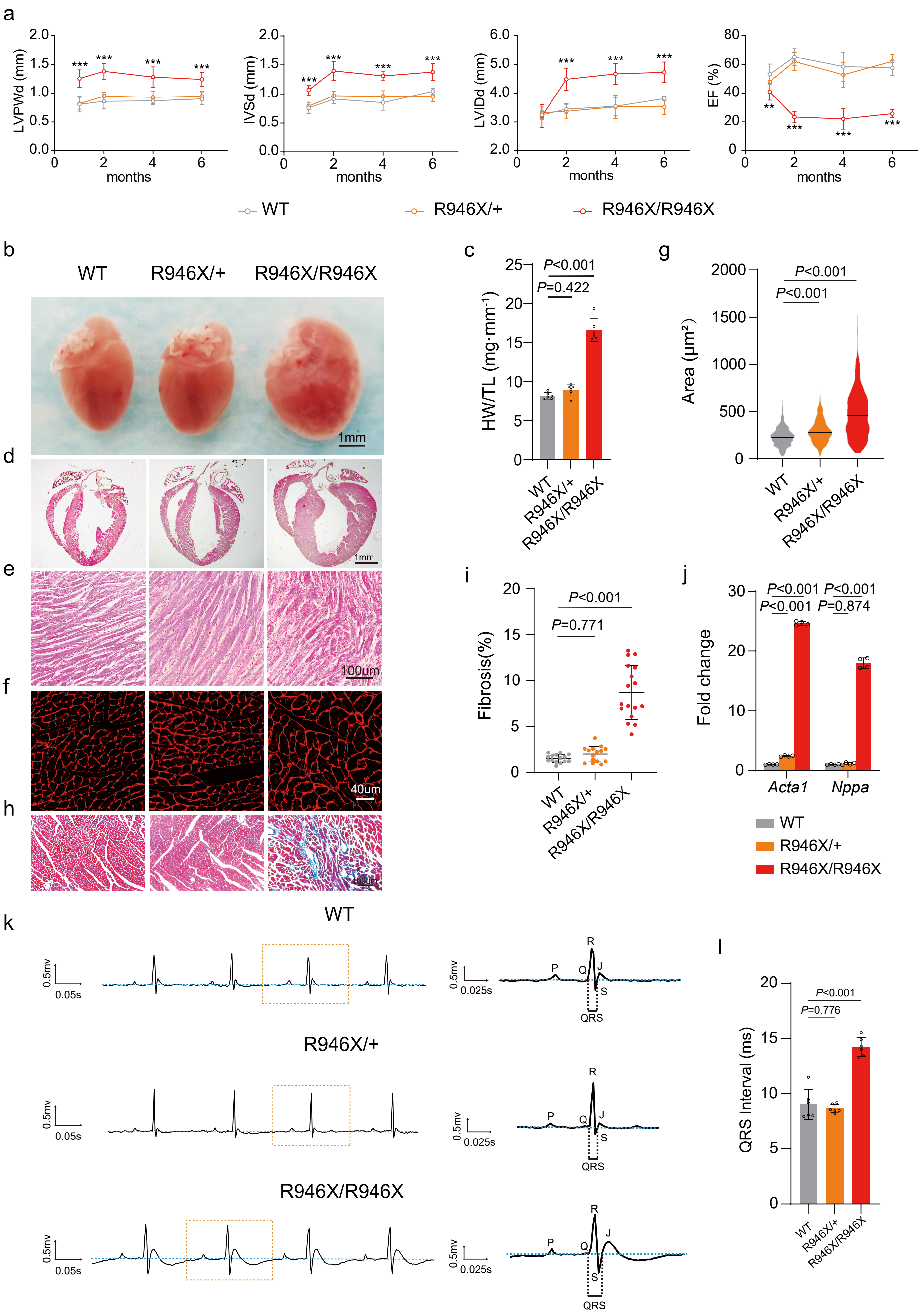

a
LVPWd (mm)
IVSd (mm)
LVIDd (mm)
EF (%)
months
***
**
WT
R946X/+
R946X/R946X
b
WT
R946X/+
R946X/R946X
1mm
c
HW/TL (mg·mm-1)
P<0.001
P=0.422
g
Area (μm²)
P<0.001
P<0.001
d
e
f
h
1mm
100um
40um
i
Fibrosis(%)
P<0.001
P=0.771
j
Fold change
P<0.001
P<0.001
P<0.001
P=0.874
Acta1
Nppa
k
WT
R946X/+
R946X/R946X
0.5mv
0.05s
0.025s
P
Q
R
S
J
QRS
l
QRS Interval (ms)
P<0.001
P=0.776

Fig. S2. ***Mybpc3***$^{\mathbf{R946X/R946X}}$ **homozygous mice developed early-onset severe cardiomyopathy with heart failure.**

a Serial M-mode echocardiography revealed the early development of cardiomyopathy in *Mybpc3*$^{R946X/R946X}$ mice. Heart function of *Mybpc3*$^{R946X/R946X}$ mice started to decline at 2 months, and the cardiac hypertrophy appeared at 1 month. LVPWd (left ventricular posterior wall thickness at diastole), IVSd (interventricular septum at diastole), LVIDd (left ventricular internal diameter at diastole), EF (ejection fraction). N ≥ 5 for each group. Data are Mean ± SD and tested with two-way ANOVA followed by Holm-Sidak's post hoc test. **: P<0.01, ***: P<0.001.

b Representative anatomy of *Mybpc3*^{wt}, *Mybpc3*$^{R946X/+}$ and *Mybpc3*$^{R946X/R946X}$ hearts 3 months old.

c Heart weight (HW) to tibia length (TL) ratio revealed a significant increase of HW in *Mybpc3*$^{R946X/R946X}$ mice at the age of 3 months. N = 6 for each group. Data are Mean ± SD and tested with one-way ANOVA followed by Tukey post hoc test. P<0.05 indicated significance.

d Coronal-section of *Mybpc3*^{wt}, *Mybpc3*$^{R946X/+}$ and *Mybpc3*$^{R946X/R946X}$ hearts at age of 3 months.

e Magnified H&E images showed the cardiac myofiber disarray in *Mybpc3*$^{R946X/R946X}$ mice at the age of 3 months.

f&g WGA staining unraveled the increased sizes of cardiomyocytes in *Mybpc3*$^{R946X/R946X}$ heart at the age of 3 months. The WGA area (**f**) representing the cardiomyocyte size were quantified with imageJ (**g**). Over 250 cells from 5 hearts in each group were calculated. Data are Mean ± SD and tested with Kruskal-Wallis test followed by Dunn's post hoc test. P<0.05 indicated significance.

h&i Masson trichome staining revealed increased fibrosis in *Mybpc3*$^{R946X/R946X}$ heart at the age of 3 months. The blue fibrotic area (**h**) from 5 tested hearts were quantified with imageJ (**i**). At least 3 slices per heart from 5 hearts were calculated for each group. Data are Mean ± SD and tested with one-way ANOVA followed by Tukey post hoc test. P<0.05 indicated significance.

j Heart failure gene expression of Acta1 and Nppa in *Mybpc3*^{wt}, *Mybpc3*$^{R946X/+}$ and *Mybpc3*$^{R946X/R946X}$ mice was quantified by RT-qPCR at the age of 3 months. N = 4 for each group. Data are Mean ± SD and tested with one-way ANOVA followed by Tukey post hoc test. P<0.05 indicated significance.

k&l Representative electrocardiograms (**k**) and QRS intervals (**l**) of *Mybpc3*^{wt}, *Mybpc3*$^{R946X/+}$ and *Mybpc3*$^{R946X/R946X}$ mice at 2 months old. *Mybpc3*$^{R946X/R946X}$ mice had prolonged QRS interval consistent with hypertrophic left ventricular.
