# Supplementary material for: Base editing effectively prevents early-onset severe cardiomyopathy in Mybpc3 mutant mice
Source: Cell Res. 2024 Feb 9;34(4):327–30. doi: 10.1038/s41422-024-00930-7 (PMC10978934; doi:10.1038/s41422-024-00930-7)
Supplement: Supplementary file 7 — Supplementary Figure S3 [file 41422_2024_930_MOESM7_ESM.pdf]

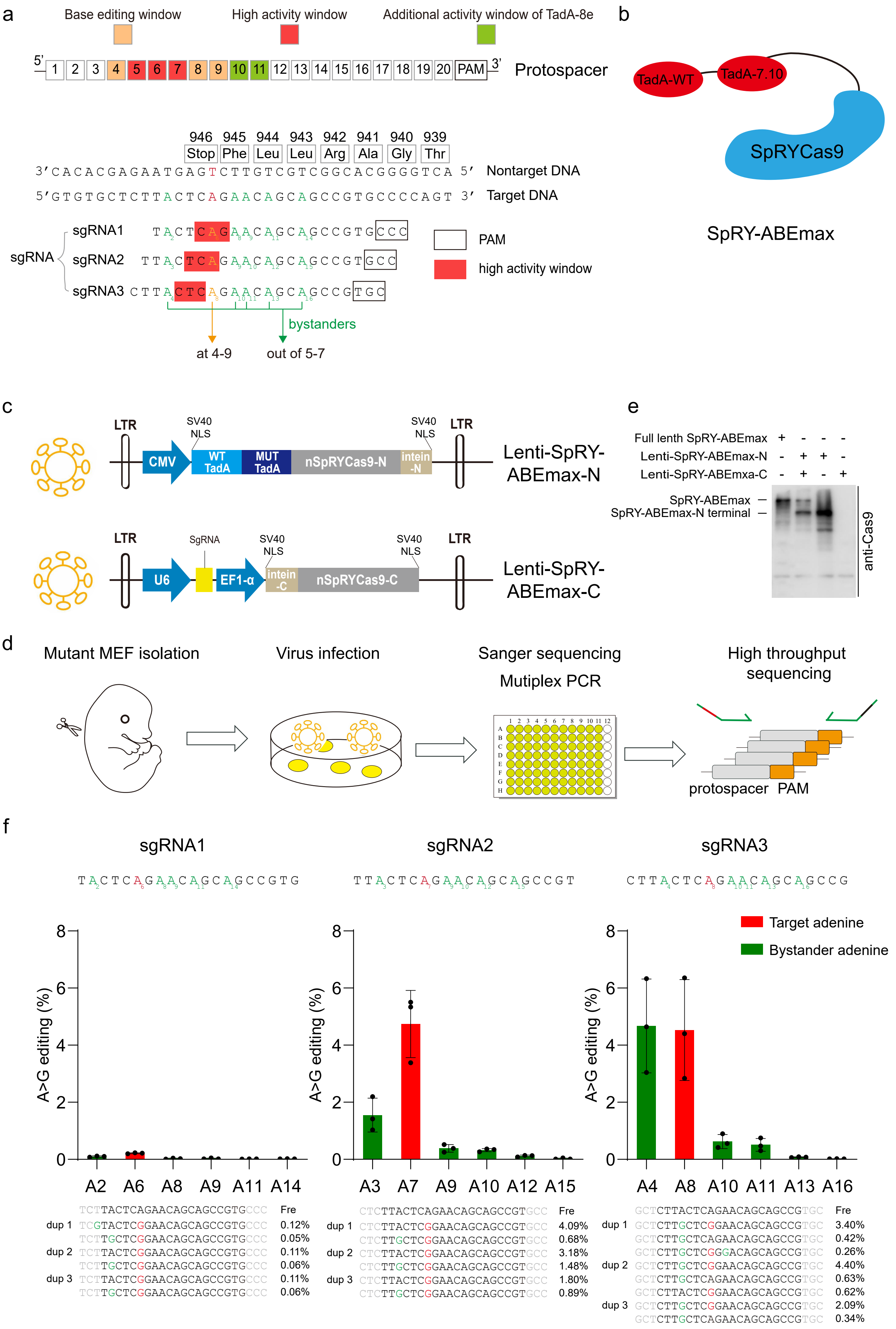
a
Base editing window
High activity window
Additional activity window of TadA-8e
Protospacer
Nontarget DNA
Target DNA
sgRNA
sgRNA1
sgRNA2
sgRNA3
PAM
high activity window
bystanders
at 4-9
out of 5-7
b
TadA-WT
TadA-7.10
SpRYCas9
SpRY-ABEmax
c
LTR
CMV
SV40 NLS
WT TadA
MUT TadA
nSpRYCas9-N
intein -N
Lenti-SpRY-ABEmax-N
U6
SgRNA
EF1-α
intein -C
nSpRYCas9-C
Lenti-SpRY-ABEmax-C
e
Full lenth SpRY-ABEmax
Lenti-SpRY-ABEmax-N
Lenti-SpRY-ABEmxa-C
SpRY-ABEmax
SpRY-ABEmax-N terminal
anti-Cas9
d
Mutant MEF isolation
Virus infection
Sanger sequencing
Mutiplex PCR
High throughput sequencing
protospacer PAM
f
sgRNA1
sgRNA2
sgRNA3
Target adenine
Bystander adenine
A>G editing (%)
A2 A6 A8 A9 A11 A14
A3 A7 A9 A10 A12 A15
A4 A8 A10 A11 A13 A16
Fre
dup 1
dup 2
dup 3

Fig. S3. Characterization of SpRY-ABEmax in MEF cells.

a Schematic diagram of base editing window of ABEmax and ABE8e and guide RNA selection.

b Schematic diagram of SpRY-ABEmax consisting SpRYCas9 nickase and two TadA.

c Schematic diagram of dual lentivirus encoding split-intein SpRY-ABEmax halves and the p.R946X-targeting sgRNAs.

d A flow chart of examining the selected guide RNAs in MEF cells by high throughput sequencing.

e Western blot showed the reconstitution of full-length SpRY-ABEmax in MEF cells 20 days after co-transduction of lentiviruses encoding SpRY-ABEmax-N&C. The assembling ratio is about 32%.

f Adenine (A) to guanine (G) transition efficiency of each A within the protospacer of sgRNA1-3 and on-target alleles frequency of each duplication in *Mybpc3*$^{R946X/R946X}$ MEF cells. The editing efficacies were assessed with high-throughput target sequencing. Data are Mean ± SD from 3 independent experiments. Dup, duplication; Fre, frequency.
