# Supplementary material for: Base editing effectively prevents early-onset severe cardiomyopathy in Mybpc3 mutant mice
Source: Cell Res. 2024 Feb 9;34(4):327–30. doi: 10.1038/s41422-024-00930-7 (PMC10978934; doi:10.1038/s41422-024-00930-7)
Supplement: Supplementary file 8 — Supplementary Figure S4 [file 41422_2024_930_MOESM8_ESM.pdf]

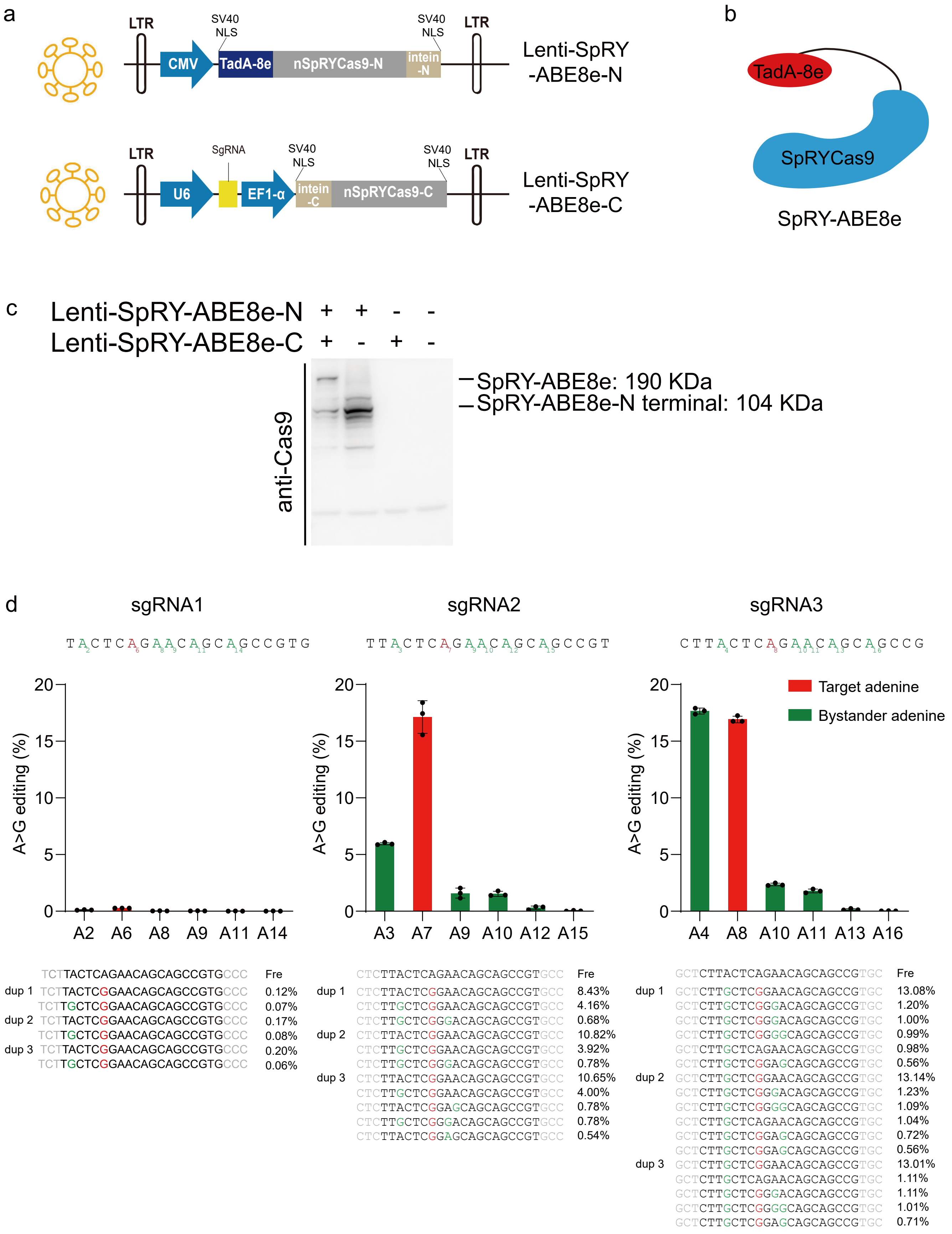
a
LTR
SV40 NLS
CMV
TadA-8e
nSpRYCas9-N
intein -N
Lenti-SpRY -ABE8e-N
U6
SgRNA
EF1-α
intein -C
nSpRYCas9-C
Lenti-SpRY -ABE8e-C
b
TadA-8e
SpRYCas9
SpRY-ABE8e
c
Lenti-SpRY-ABE8e-N + + - -
Lenti-SpRY-ABE8e-C + - + -
anti-Cas9
SpRY-ABE8e: 190 KDa
SpRY-ABE8e-N terminal: 104 KDa
d
sgRNA1
TACTCAGAACAGCAGCCGTG
sgRNA2
TTACTCAGAACAGCAGCCGT
sgRNA3
CTTACTCAGAACAGCAGCCG
Target adenine
Bystander adenine
A>G editing (%)
A2 A6 A8 A9 A11 A14
A3 A7 A9 A10 A12 A15
A4 A8 A10 A11 A13 A16
TCTTACTCAGAACAGCAGCCGTGCCC Fre
dup 1 TCTTACTCGGAACAGCAGCCGTGCCC 0.12%
TCTTGCTCGGAACAGCAGCCGTGCCC 0.07%
dup 2 TCTTACTCGGAACAGCAGCCGTGCCC 0.17%
TCTTGCTCGGAACAGCAGCCGTGCCC 0.08%
dup 3 TCTTACTCGGAACAGCAGCCGTGCCC 0.20%
TCTTGCTCGGAACAGCAGCCGTGCCC 0.06%
CTCTTACTCAGAACAGCAGCCGTGCC Fre
dup 1 CTCTTACTCGGAACAGCAGCCGTGCC 8.43%
CTCTTGCTCGGAACAGCAGCCGTGCC 4.16%
CTCTTGCTCGGGACAGCAGCCGTGCC 0.68%
dup 2 CTCTTACTCGGAACAGCAGCCGTGCC 10.82%
CTCTTGCTCGGAACAGCAGCCGTGCC 3.92%
CTCTTGCTCGGGACAGCAGCCGTGCC 0.78%
dup 3 CTCTTACTCGGAACAGCAGCCGTGCC 10.65%
CTCTTGCTCGGAACAGCAGCCGTGCC 4.00%
CTCTTACTCGGAGCAGCAGCCGTGCC 0.78%
CTCTTGCTCGGGACAGCAGCCGTGCC 0.78%
CTCTTACTCGGAGCAGCAGCCGTGCC 0.54%
GCTCTTACTCAGAACAGCAGCCGTGC Fre
dup 1 GCTCTTGCTCGGAACAGCAGCCGTGC 13.08%
GCTCTTGCTCGGGACAGCAGCCGTGC 1.20%
GCTCTTGCTCGGGACAGCAGCCGTGC 1.00%
GCTCTTGCTCGGGGCAGCAGCCGTGC 0.99%
GCTCTTGCTCAGAACAGCAGCCGTGC 0.98%
GCTCTTGCTCGGAGCAGCAGCCGTGC 0.56%
dup 2 GCTCTTGCTCGGAACAGCAGCCGTGC 13.14%
GCTCTTGCTCGGGACAGCAGCCGTGC 1.23%
GCTCTTGCTCGGGGCAGCAGCCGTGC 1.09%
GCTCTTGCTCAGAACAGCAGCCGTGC 1.04%
GCTCTTGCTCGGAGCAGCAGCCGTGC 0.72%
GCTCTTGCTCGGAGCAGCAGCCGTGC 0.56%
dup 3 GCTCTTGCTCGGAACAGCAGCCGTGC 13.01%
GCTCTTGCTCAGAACAGCAGCCGTGC 1.11%
GCTCTTGCTCGGGACAGCAGCCGTGC 1.11%
GCTCTTGCTCGGGGCAGCAGCCGTGC 1.01%
GCTCTTGCTCGGAGCAGCAGCCGTGC 0.71%

Fig. S4. *In vitro* characterization of SpRY-ABE8e.

a Schematic diagram of dual lentivirus encoding split-intein SpRY-ABE8e halves and the p.R946X-targeting sgRNAs.

b Schematic diagram of SpRY-ABE8e consisting of SpRYCas9 nickase and TadA-8e-V106W.

c Western blot showed N and C part of SpRY-ABE8e assembled in MEF cells.

d A to G editing efficiency of each A within target protospacer and on-target alleles frequency of each duplication in *Mybpc3*$^{R946X/R946X}$ MEF cells 20 days post transfection as revealed by target HT-seq. Data are Mean ± SD from 3 independent experiments. Dup, duplication; Fre, frequency.
