# Supplementary material for: Base editing effectively prevents early-onset severe cardiomyopathy in Mybpc3 mutant mice
Source: Cell Res. 2024 Feb 9;34(4):327–30. doi: 10.1038/s41422-024-00930-7 (PMC10978934; doi:10.1038/s41422-024-00930-7)
Supplement: Supplementary file 9 — Supplementary Figure S5 [file 41422_2024_930_MOESM9_ESM.pdf]

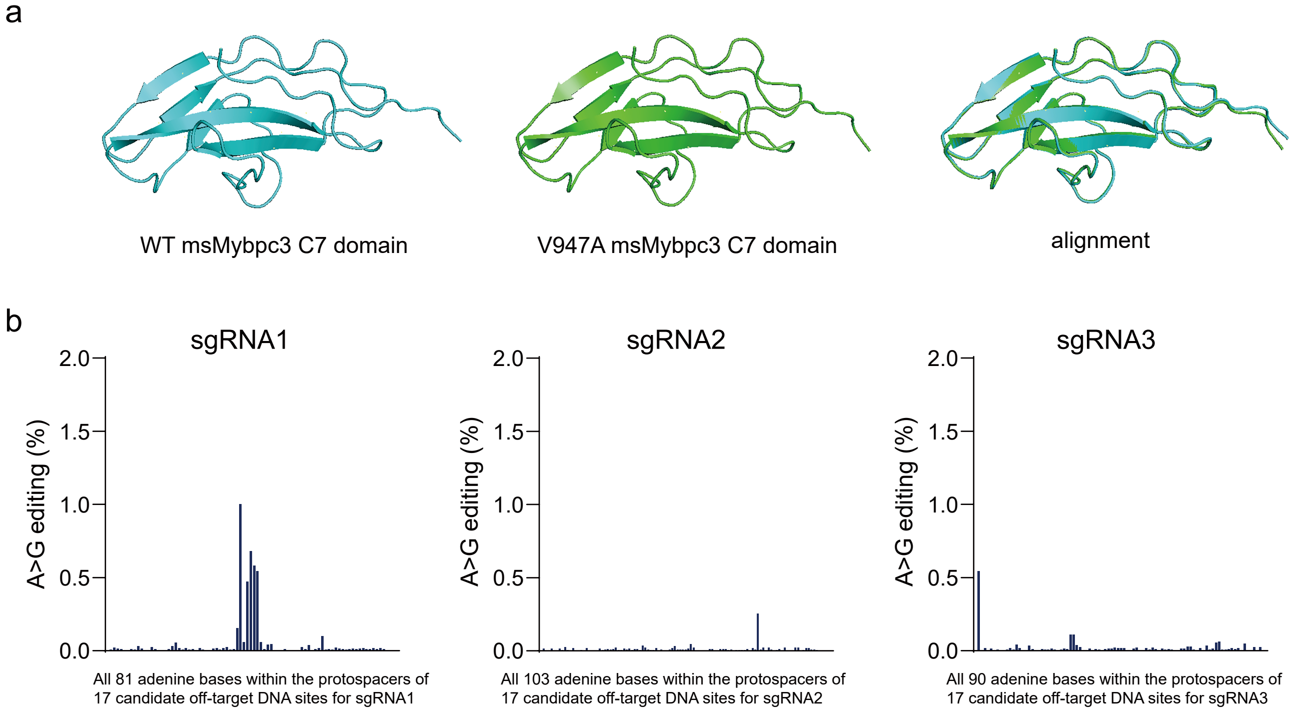

Fig. S5. Off-target analysis of SpRY-ABE8e in MEF cells.

a Alignment of p.V947A MYBPC3 C7 domain predicted by AlphaFold2 to WT MYBPC3 C7 domain showed the p.V947A substitution didn't change MYBPC3 structure.

b Off-target analysis using HT sequencing for 17 most potential off-target loci identified by Cas-OFFinder for each sgRNA in *Mybpc3*$^{R946X/R946X}$ MEF cells.
