# Supplementary material for: Base editing effectively prevents early-onset severe cardiomyopathy in Mybpc3 mutant mice
Source: Cell Res. 2024 Feb 9;34(4):327–30. doi: 10.1038/s41422-024-00930-7 (PMC10978934; doi:10.1038/s41422-024-00930-7)
Supplement: Supplementary file 10 — Supplementary Figure S6 [file 41422_2024_930_MOESM10_ESM.pdf]

a

sgRNA1

Target	Chromosome	Position	Direction	Gene
OT-NR-1	chr18	68933396	+	Intergenic
OT-NR-2	chr7	142894083	-	Th
OT-NR-3	chr13	29408631	+	Cdkal intron
OT-NR-4	chr16	51530354	+	Intergenic
OT-NR-5	chr14	57264185	-	Intergenic
OT-NR-6	chr10	63641730	+	Ctnna3 intron
OT-NR-7	chr1	113771229	-	Intergenic
OT-NR-8	chr2	80856190	-	Intergenic
OT-NY-1	chr10	12190633	+	Intergenic
OT-NY-2	chr1	193951646	-	Intergenic
OT-NY-3	chr3	49769983	-	Intergenic
OT-NY-4	chr3	38454310	-	Ankrd50
OT-edited-1	chr5	37331480	-	Evc intron
OT-edited-2	chrX	86317402	+	Intergenic
OT-edited-3	chr4	22479221	+	Intergenic
OT-edited-4	chr10	60657190	-	Cdh23 intron
OT-edited-5	chr19	48259917	+	Sorcs3 intron

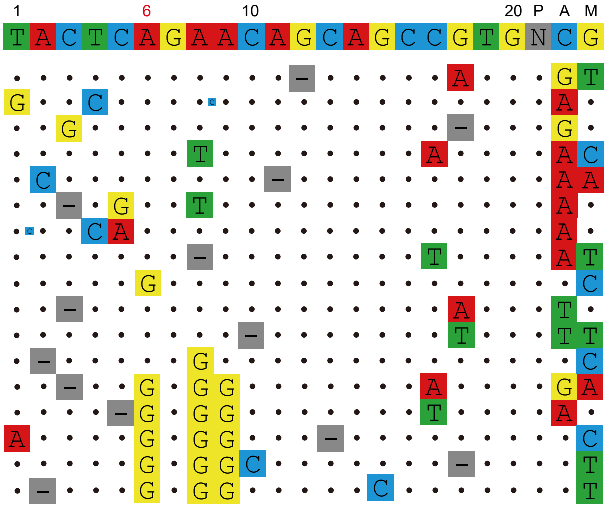

sgRNA2

Target	Chromosome	Position	Direction	Gene
OT-NR-1	chr18	50470961	+	Intergenic
OT-NR-2	chr2	119251308	+	Intergenic
OT-NR-3	chrX	36261484	-	Intergenic
OT-NR-4	chr12	8344932	-	Intergenic
OT-NR-5	chr5	90087429	-	Intergenic
OT-NR-6	chr15	39813986	+	Dpys intron
OT-NR-7	chr9	17833299	+	Intergenic
OT-NR-8	chr1	13615994	-	Intergenic
OT-NY-1	chr17	50799263	+	Tbc1d5 intron
OT-NY-2	chr11	119172449	-	Tbc1d16 intron
OT-NY-3	chr6	45448548	+	Cntnap2 intron
OT-NY-4	chr3	97862587	+	Pde4dip intron
OT-edited-1	chr8	83428317	+	Clgn 3' UTR
OT-edited-2	chr6	94694431	+	Lrig1 intron
OT-edited-3	chr5	30182580	+	Hadhb intron
OT-edited-4	chr15	36313390	+	Intergenic
OT-edited-5	chr2	74491529	+	Intergenic

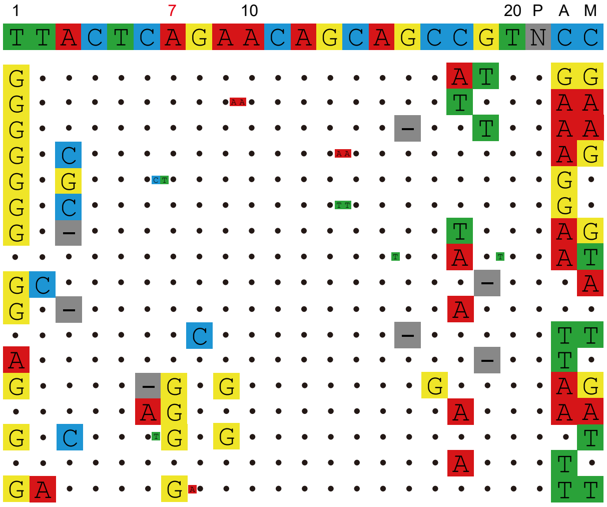

sgRNA3

Target	Chromosome	Position	Direction	Gene
OT-NR-1	chr5	48010412	-	Slit2 intron
OT-NR-2	chr8	72475509	-	intergenic
OT-NR-3	chr15	79290746	+	Pla2g6 intron
OT-NR-4	chr12	84206788	-	Elmasan1 intron
OT-NR-5	chr12	91692056	-	Ston2 intron
OT-NR-6	chr2	35412735	-	Ggta1 intron
OT-NR-7	chr18	7693361	+	intergenic
OT-NR-8	chr13	20373083	+	Elmo1
OT-NY-1	chr7	113566703	-	Far1 intron
OT-NY-2	chr1	165472486	-	Mpc2 intron
OT-NY-3	chr11	107755827	-	Cacng4 intron
OT-NY-4	chr2	121410935	-	Catsper2 intron
OT-edited-1	chr5	102890108	-	Arhgap24 intron
OT-edited-2	chr9	94820702	+	Slc9a9 intron
OT-edited-3	chr12	85225396	-	Eif2b2 intron
OT-edited-4	chr3	133527132	-	Tet2 intron
OT-edited-5	chr12	72794491	-	Ppm1a 3' UTR

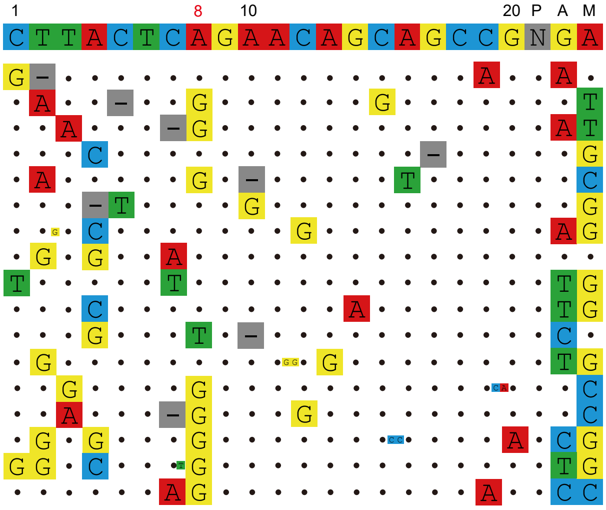

Fig. S6. Off-target sites for each sgRNA with SpRY-ABE8e.

a Genomic loci (left) and sequences of 17 candidate off-target (OT) sites aligned to the protospacer of each sgRNA (right). Position indicates the start of the sequence. Nucleotides matching the protospacer are shown as a dot. Nucleotides differing from the protospacer are directly labeled in each site. DNA bulges are indicated by additional nucleotides between normal nucleotides. RNA bulges are indicated by a transverse line. NR indicated OT sites with NR PAM; NY indicated OT sites with NY PAM; OT-edited indicated OT sites with edited protospacer.
