# Supplementary material for: Base editing effectively prevents early-onset severe cardiomyopathy in Mybpc3 mutant mice
Source: Cell Res. 2024 Feb 9;34(4):327–30. doi: 10.1038/s41422-024-00930-7 (PMC10978934; doi:10.1038/s41422-024-00930-7)
Supplement: Supplementary file 11 — Supplementary Figure S7 [file 41422_2024_930_MOESM11_ESM.pdf]

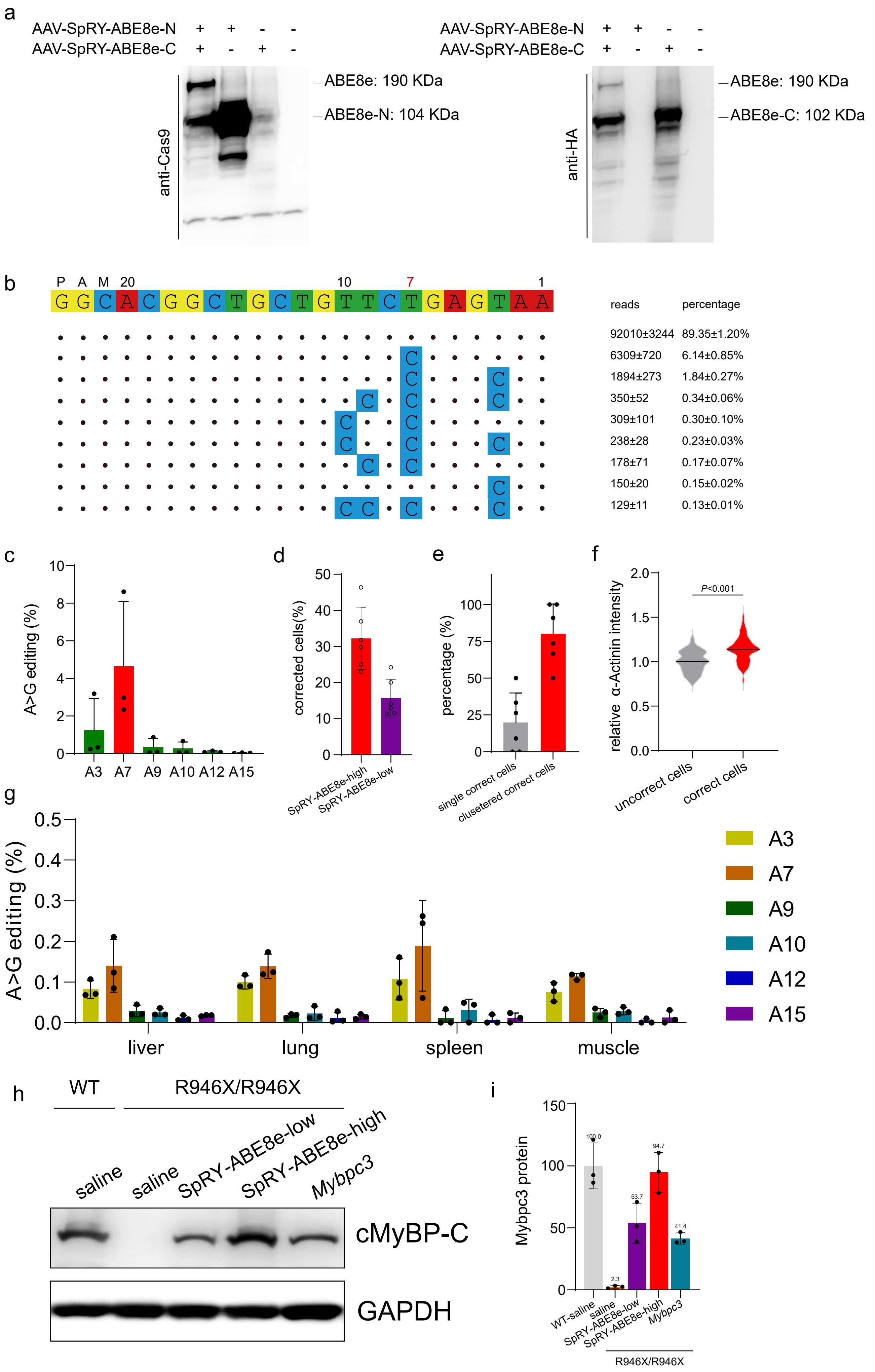
a
AAV-SpRY-ABE8e-N + + - -
AAV-SpRY-ABE8e-C + - + -
anti-Cas9
ABE8e: 190 KDa
ABE8e-N: 104 KDa
AAV-SpRY-ABE8e-N + + - -
AAV-SpRY-ABE8e-C + - + -
anti-HA
ABE8e: 190 KDa
ABE8e-C: 102 KDa
b
P A M 20 10 7 1
G G C A C G G C T G C T G T T C T G A G T A A
reads percentage
92010±3244 89.35±1.20%
6309±720 6.14±0.85%
1894±273 1.84±0.27%
350±52 0.34±0.06%
309±101 0.30±0.10%
238±28 0.23±0.03%
178±71 0.17±0.07%
150±20 0.15±0.02%
129±11 0.13±0.01%
c
A>G editing (%)
A3 A7 A9 A10 A12 A15
d
corrected cells(%)
SpRY-ABE8e-high
SpRY-ABE8e-low
e
percentage (%)
single correct cells
clusetered correct cells
f
relative α-Actinin intensity
P<0.001
uncorrect cells
correct cells
g
A>G editing (%)
liver
lung
spleen
muscle
A3
A7
A9
A10
A12
A15
h
WT
R946X/R946X
saline
saline
SpRY-ABE8e-low
SpRY-ABE8e-high
Mybpc3
cMyBP-C
GAPDH
i
Mybpc3 protein
100.0
2.3
53.7
94.7
41.4
WT-saline
saline
SpRY-ABE8e-low
SpRY-ABE8e-high
Mybpc3
R946X/R946X

Fig. S7. *In vivo* characterization of AAV-SpRY-ABE8e.

a Western blot showed that SpRY-ABE8e N terminal assembled with C terminal to constitute full-length SpRY-ABE8e in mice heart 14 days post AAV injection.

b On-target alleles frequency of high-dose-SpRY-ABE8e with sgRNA2 in *Mybpc3*$^{R946X/R946X}$ hearts 6 months post injection as revealed by targeted HT-seq. N = 3. Data are Mean ± SD.

c Base correcting efficiency in the heart 6 months after low-dose-SpRY-ABE8e treatment. N = 3. Data: Mean ± SD.

d Quantify the percentage of MYBPC3-recovery cardiomyocytes at 6 months after SpRY-ABE8e treatment. N = 6. Data: Mean ± SD.

e IF staining revealed ABE corrected cells are more in cluster form than in single form. Correct cells next to other correct cells are counted as clustered correct cells while the others were counted as single correct cells. 3 slides for each mouse from 6 high-dose SpRY-ABE8e treated mice are counted.

f Higher expression level of α-Actinin in corrected cells than in uncorrected cells indicate recovered gene expression in corrected cells. The expression level was quantified by the fluorescence intensity of α-Actinin in total 18 slides from 6 high-dose SpRY-ABE8e treated mice. Data are tested with one-way ANOVA followed by Tukey post hoc test. $P<0.05$ indicated significance.

g On-target editing efficiency in liver, lung, spleen, muscle was much lower than in heart after high-dose-SpRY-ABE8e treatment as revealed by HTS. N = 3 samples. Data are Mean ± SD.

h&i MYBPC3 protein restoration 6 months after AAV infection as revealed by western blot. The level of protein expression indicated by blot intensity (**h**) was quantified with imageJ (**i**). N = 3 for each group. Data: Mean ± SD.
