# Supplementary material for: Base editing effectively prevents early-onset severe cardiomyopathy in Mybpc3 mutant mice
Source: Cell Res. 2024 Feb 9;34(4):327–30. doi: 10.1038/s41422-024-00930-7 (PMC10978934; doi:10.1038/s41422-024-00930-7)
Supplement: Supplementary file 13 — Supplementary Figure S9 [file 41422_2024_930_MOESM13_ESM.pdf]

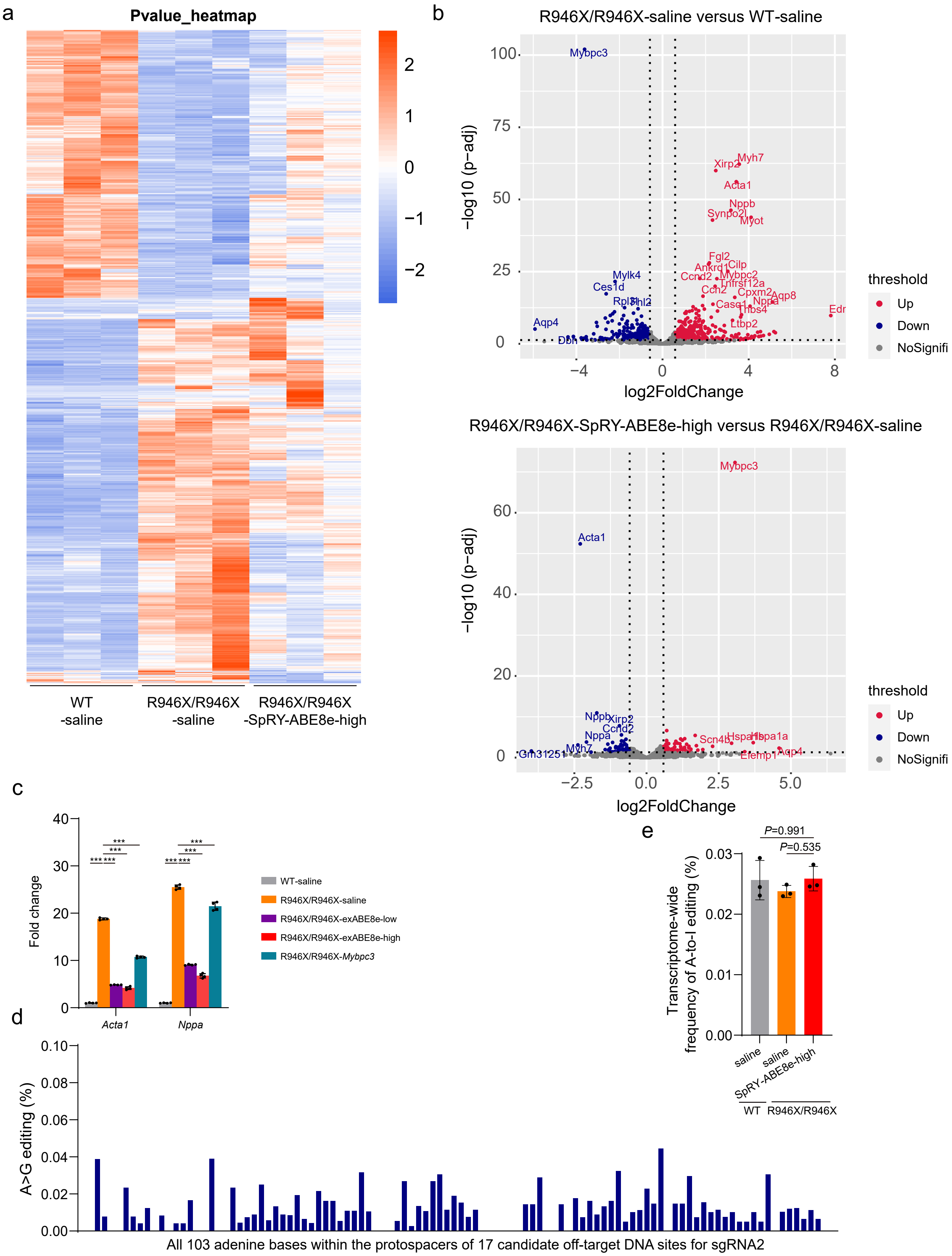
a
Pvalue_heatmap
2
1
0
−1
−2
WT
-saline
R946X/R946X
-saline
R946X/R946X
-SpRY-ABE8e-high
b
R946X/R946X-saline versus WT-saline
−log10 (p−adj)
log2FoldChange
threshold
Up
Down
NoSignifi
Mybpc3
Myh7
Xirp2
Acta1
Nppb
Synpo2l
Myot
Fgl2
Cilp
Ankrd1
Ccnd2
Mybpc2
Tnfrsf12a
Ccn2
Cpxm2
Casq1
Nppa
Aqp8
Thbs4
Ltbp2
Edr
Mylk4
Ces1d
Rpl3l
Fhl2
Aqp4
Dbh
R946X/R946X-SpRY-ABE8e-high versus R946X/R946X-saline
Mybpc3
Acta1
Nppb
Xirp2
Ccnd2
Nppa
Myh7
Gm31251
Scn4b
Hspa1b
Hspa1a
Efemp1
Aqp4
c
Fold change
Acta1
Nppa
WT-saline
R946X/R946X-saline
R946X/R946X-exABE8e-low
R946X/R946X-exABE8e-high
R946X/R946X-Mybpc3
d
A>G editing (%)
All 103 adenine bases within the protospacers of 17 candidate off-target DNA sites for sgRNA2
e
Transcriptome-wide
frequency of A-to-I editing (%)
P=0.991
P=0.535
saline
saline
SpRY-ABE8e-high
WT
R946X/R946X

Fig. S9. Genomic and transcriptomic analysis of AAV-SpRY-ABE8e-treated hearts.

a Heat map of 727 differentially expressed genes in hierarchy clusters revealed by RNA-seq. High-dose SpRY-ABE8e changed transcriptomic profile of *Mybpc3*$^{R946X/R946X}$ to wildtype heart.

b Volcano plot showed the identified DEGs in saline-treated *Mybpc3*$^{R946X/R946X}$ mice versus *Mybpc3*^{wt} mice (left), and in high-dose-SpRY-ABE8e-treated *Mybpc3*$^{R946X/R946X}$ mice versus saline-treated *Mybpc3*$^{R946X/R946X}$ mice (right). N = 3 for each group.

c Heart failure gene expression of *Acta1* and *Nppa* declined in *Mybpc3*$^{R946X/R946X}$ mice after AAV treatment especially in high-dose SpRY-ABE8e treated group as quantified by RT-qPCR 6 months post injection. N = 4 for each group. Data are Mean ± SD and tested with two-way ANOVA followed by Tukey post hoc test. ***: P<0.001.

d Off-target analysis of sgRNA2 using HT-seq for 17 most potential off-target loci containing 103 adenine bases identified by Cas-OFFinder in *Mybpc3*$^{R946X/R946X}$ heart 6 months after high-dose-SpRY-ABE8e treatment. The editing rates at all tested off-target sites were below 0.05%.

e Adenine-to-inosine (A-to-I) transition was calculated from RNA-seq data. N=3 for each group. Data are Mean ± SD and tested with one-way ANOVA followed by Tukey post hoc test. P<0.05 indicated significance.
