# Supplementary material for: Base editing effectively prevents early-onset severe cardiomyopathy in Mybpc3 mutant mice
Source: Cell Res. 2024 Feb 9;34(4):327–30. doi: 10.1038/s41422-024-00930-7 (PMC10978934; doi:10.1038/s41422-024-00930-7)
Supplement: Supplementary file 12 — Supplementary Figure S8 [file 41422_2024_930_MOESM12_ESM.pdf]

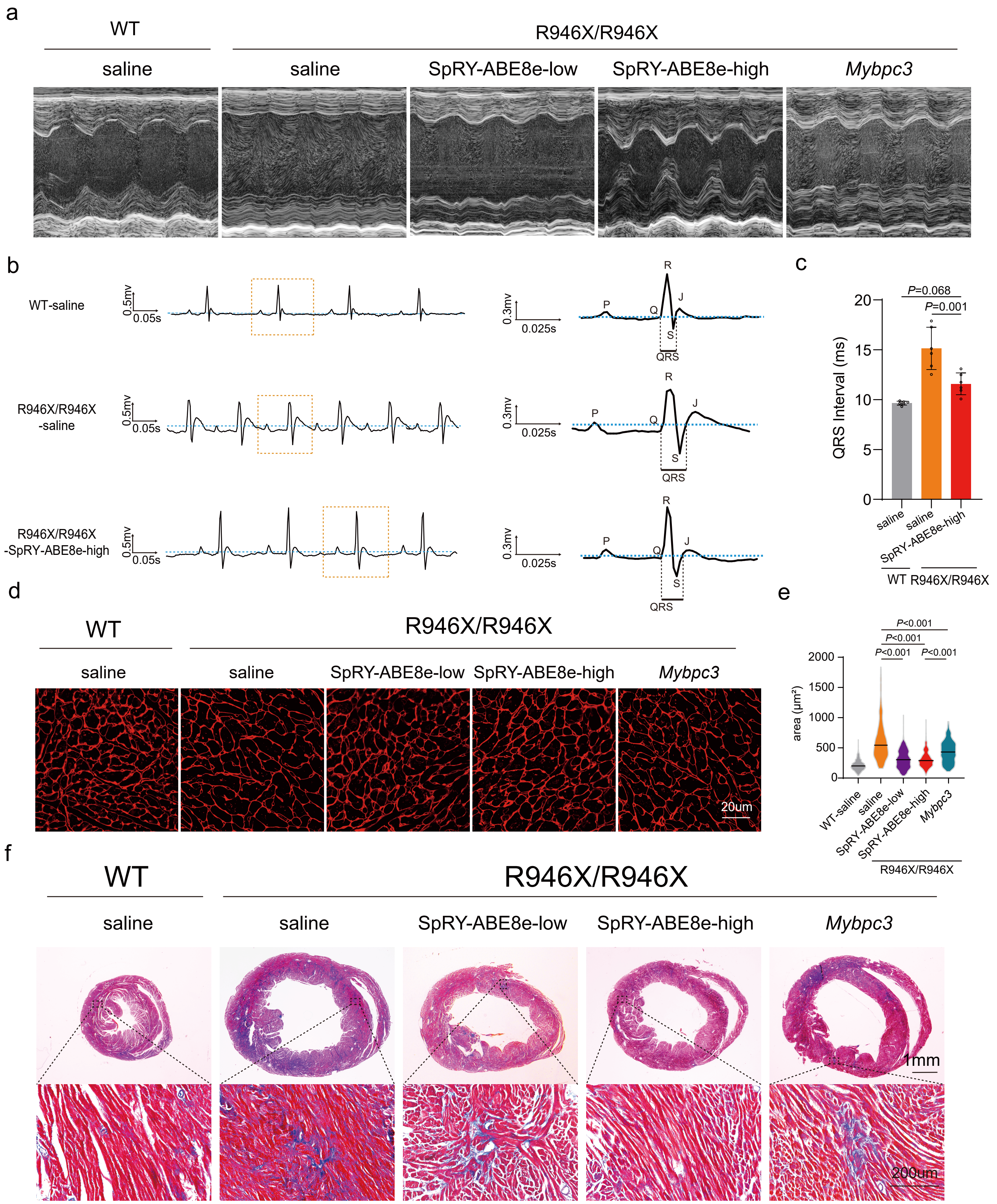

a
WT
R946X/R946X
saline
saline
SpRY-ABE8e-low
SpRY-ABE8e-high
Mybpc3
b
WT-saline
R946X/R946X
-saline
R946X/R946X
-SpRY-ABE8e-high
0.5mv
0.05s
0.3mv
0.025s
P
Q
R
S
J
QRS
c
P=0.068
P=0.001
QRS Interval (ms)
0
5
10
15
20
saline
saline
SpRY-ABE8e-high
WT
R946X/R946X
d
WT
R946X/R946X
saline
saline
SpRY-ABE8e-low
SpRY-ABE8e-high
Mybpc3
20um
e
P<0.001
P<0.001
P<0.001
P<0.001
area (μm²)
0
500
1000
1500
2000
WT-saline
saline
SpRY-ABE8e-low
SpRY-ABE8e-high
Mybpc3
R946X/R946X
f
WT
R946X/R946X
saline
saline
SpRY-ABE8e-low
SpRY-ABE8e-high
Mybpc3
1mm
200um

Fig. S8. SpRY-ABE8e prevented cardiomyopathy in a dose-dependent manner *in vivo*.

a Representative M-echocardiography at 6 months after AAV injection. N = 5-6 mice for each group.

b&c Electrocardiograms (**b**) and QRS intervals (**c**) of saline- or virus-treated mice 2 months post injection. N = 6 for each group. Data are Mean ± SD and tested with one-way ANOVA followed by Tukey post hoc test. $P<0.05$ indicated significance.

d&e WGA staining showed AAV-SpRY-ABE8e in either low or high dose and AAV-*Mybpc3* reduced the *Mybpc3*$^{R946X/R946X}$ cardiomyocyte size compared with saline. WGA area (**d**) representing the cardiomyocyte size were quantified using Image J (**e**). Over 150 cells from 5 hearts in each group were calculated. Data are tested with Kruskal-Wallis test followed by Dunn's post hoc test. $P<0.05$ indicated significance.

f Whole transverse cross-sections of Masson trichrome stained hearts revealed reduced fibrosis in all AAV-treated groups.
